# A Japanese-Dietitian Prompt Systematically Shifts Portion Estimates in LLM-Based Nutrient Estimation from Food Images: A Multi-Dataset, Multi-Model Study

**DOI:** 10.3390/nu18172892

**Published:** 2026-09-03

**Authors:** Shinichi Nakagawa, Akira Yamamoto

**Affiliations:** 1 Research Institute of Info-Communication Medicine (RinCOM), Tokyo 184-0004, Japan; 2 Faculty of Health Data Science, Juntendo University, Tokyo 113-8421, Japan

**Keywords:** vision-language models, dietary assessment, portion size, persona prompting, prompt engineering, nutrient estimation, food images

## Abstract

**Background/Objectives:** In food-image nutrient estimation with vision-language models (VLMs), portion size is a dominant source of error, yet how a prompt shifts the estimated amount—and in which direction—remains uncharacterized. We tested whether a Japanese-dietitian prompt acts as a systematic, directional influence on a model’s quantity estimates and what an explicit magnitude instruction does by comparison. **Methods:** Six VLMs from three vendors were evaluated on two datasets with contrasting portion regimes—NutriImage (Japanese cafeteria dishes; dietitian-calculated ground truth) and SNAPMe (US meal photographs)—under persona conditions (none, Japanese-dietitian, US-dietitian) crossed with two portion-specification levels, with five additional prompt-control conditions, image-level paired statistics with bootstrap confidence intervals, interaction tests, equivalence tests, and a repeated-call variability analysis. **Results:** The Japanese-dietitian prompt lowered predicted energy in all six models and both datasets (persona main effect *p* < 10^−94^), approximately preserving predicted macronutrient composition in relative terms; the US prompt produced only small, sign-inconsistent changes. The effect was not reproduced by an explicit “assume smaller portions” instruction, which shifted estimates further but far less consistently, whereas an “assume larger portions” instruction was followed almost uniformly by four of the six models, with both OpenAI models largely insensitive to explicit magnitude instructions in either direction. Accuracy consequences were dataset-dependent: error decreased on the small-portion dataset (up to ~20 MedAPE points) and was statistically equivalent (±5-point margin) on the larger-portion dataset in 11 of 12 model × portion cells. All principal effects were confirmed in a unified analysis that randomizes model identity over a pooled image set (*n* = 2159 independent images; Holm-corrected; robust across 1000 random re-assignments). **Conclusions:** A Japanese-dietitian prompt acts as a consistent downward influence on VLM quantity estimates that is distinct from explicit downscaling instructions; its accuracy value is domain-specific and requires validation on the target domain before any practical use.

## 1. Introduction

Vision-language models (VLMs) are increasingly applied to dietary assessment, estimating nutrient content directly from a photograph as a low-burden alternative to self-report [1,2]. In prior work, we showed that both prompt design and model selection substantially affect estimation accuracy in this task [1]. However, that work characterized *how much* accuracy varied, not *in which direction* a given prompt moves the estimate—the question we take up here.

In vision-based dietary assessment, portion-size estimation is a dominant source of error: because a single scaling error propagates to every nutrient, mis-estimated amount rather than mis-identified composition accounts for much of the variance in energy and macronutrient predictions [1,3,4]. Yet the systematic *direction* of this error under a given prompt has not been characterized; our prior work noted calibration of directional bias as an open question [1]. Interventions that adjust a model’s implicit sense of *how much food is present* are therefore of particular practical value when ground-truth portion weight is unavailable.

Persona prompting has been studied largely as a source of *social and cultural* bias: nationality personas, for example, systematically shift how models evaluate nations and attribute emotions [5,6]. Separately, LLMs are known to show anchoring-like distortions in *numerical* estimation [7,8]. Whether a persona label can act as a systematic operator on a model’s *quantitative prior*—rather than on its social judgments—has, to our knowledge, not been examined.

Whereas Zheng et al. report that persona effects on objective tasks are largely inconsistent and hard to predict [9], we document a persona effect that is *directional and consistent* across all six tested models (three vendors) and both datasets: a Japanese-dietitian persona systematically lowers predicted portions, an effect consistent with a downward shift of the model’s portion prior.

In this study, using two datasets with contrasting portion regimes and six models across three vendors, we (i) characterize this directional persona effect and its JP–US asymmetry, (ii) separate its directional signature from its accuracy consequence, and (iii) show the effect to be approximately composition-neutral and robust to removing scene context by cropping. We hypothesized, following an initial exploratory observation, that the Japanese-dietitian prompt would shift estimated amounts downward relative to the none and US-dietitian conditions; the analyses reported here were specified after that observation (Section 4).

## 2. Materials and Methods

### 2.1. Datasets

**Figure 1:**
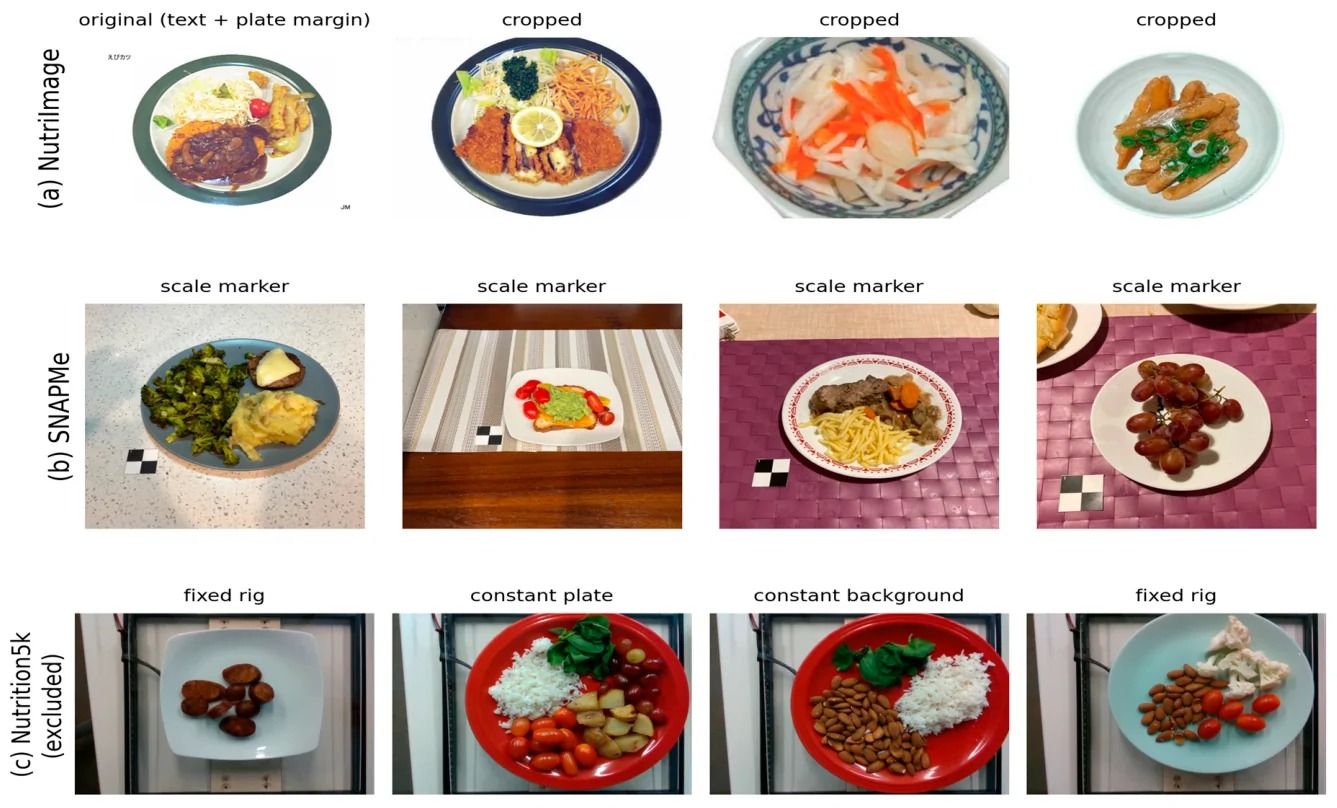
Representative images from the datasets considered. (**a**) NutriImage: an original image and three examples cropped to the dish. The original panel retains the burned-in Japanese text that the cropping procedure removes—here the dish name (ebi-katsu, a breaded fried-prawn cutlet) and a menu code—shown to illustrate the pre-processing step; no such text remains in the images used for analysis. (**b**) SNAPMe: US meal photographs, each including a checkerboard scale marker. (**c**) Nutrition5k: recorded under a fixed overhead rig with a constant plate and background; shown for comparison but not used in this study. The persona analysis was conducted on NutriImage (cropped) and SNAPMe only. SNAPMe images in (**b**) are reproduced from Larke et al. [2] and Nutrition5k images in (**c**) from Thames et al. [10], both under the Creative Commons Attribution 4.0 International (CC BY 4.0) license; NutriImage images in (**a**) are the authors’ own.

The NutriImage set used here differs from our prior work [1] in three respects: burned-in text was removed by cropping to the GPT-4o–detected dish bounding box (rather than by mask-filling), the ground truth was rebuilt from the source recipe tables, and the resulting evaluation set comprises 899 dish images, of which 887 with linked ground truth enter the quantitative analyses (Figure 1; sample-size flow in Figure S1). The dataset is archived on Zenodo (Concept DOI 10.5281/zenodo.21944881; version 1.1, used in this study, 10.5281/zenodo.21961032).

The photographs originate from the “Health and Nutrition Information” project: dishes served at the Kyoto University co-op cafeteria were photographed in 2002–2005, and the ground-truth values were calculated by registered dietitians from the ingredient-level recipe of each served dish, using the Standard Tables of Food Composition in Japan (Fifth Revised Edition, 2000), together with the total served weight. The images depict prepared food only—no persons are shown—and burned-in text present in the originals was removed by the cropping procedure described above. The images are the authors’ own; the dataset has been curated by the corresponding author since the original project and is archived under the authors’ copyright, with provenance, file formats, and access conditions documented in the README bundled with the Zenodo record.

Because our objective is to estimate the ground-truth portion present in each photograph, the datasets must present naturally varying amounts, backgrounds, and serving vessels. Nutrition5k [10], although large and precisely weighed, was recorded under a fixed overhead rig with a constant background and plate; this uniform setting removes the visual variation in portion context that our question depends on, so it was not used for the persona analysis. We therefore evaluated NutriImage (cropped) and SNAPMe (snapme_db_09Dec2022; Ag Data Commons, DOI 10.15482/USDA.ADC/1528346) [2], the latter providing US meal photographs that include a checkerboard scale marker (Figure 1). Ground-truth distributions and portion-weight statistics for both datasets are summarized in Supplementary Table S7.

Table 1 profiles the two datasets. They differ deliberately and on multiple dimensions: NutriImage contributes small, single-dish servings of everyday Japanese cafeteria food, whereas SNAPMe contributes whole, participant-captured U.S. eating occasions, roughly 1.6 times larger in median ground-truth energy and 1.8 times heavier in median portion weight. Both, however, consist of ordinary, everyday food—the kind of meals people routinely eat at home, buy ready-made, or order in casual restaurants—rather than laboratory-standardized dishes.

Table 2 summarizes the baseline residuals of the six models. On average, the models over-estimate the caloric nutrients on NutriImage and under-estimate them on SNAPMe, with the SNAPMe under-estimation concentrated in the larger meals (negative means with near-zero medians); salt alone shows mean under-estimation on both datasets. These baseline residuals are the reference for the accuracy consequences analyzed in Section 3.4.

### 2.2. Models

We tested six vision-language models spanning three vendors: Claude Haiku 4.5 and Sonnet 4.6 (Anthropic, San Francisco, CA, USA), GPT-4o and GPT-4o-mini (OpenAI, San Francisco, CA, USA), and Gemini Flash and Pro (Google, Mountain View, CA, USA).

### 2.3. Prompts and Experimental Conditions

**Base task instruction (all conditions).** Every query used a common instruction asking the model to estimate the five mandatory nutrients (energy, protein, fat, carbohydrate, salt equivalent) for the food shown in the image and to return them as a structured object. The base instruction contained neither a persona clause nor any portion cue, defining a neutral baseline onto which the two experimental factors were added independently.

**Factor 1—Persona (none/JP/US).** We prepended one of three persona clauses to the base instruction. The *none* condition added no persona and served as the control. The *JP* condition framed the model as a Japanese registered dietitian; the *US* condition, as a US registered dietitian. The exact clauses were: JP—“You are a Japanese registered dietitian (kanri-eiyoshi). You are deeply familiar with standard single-serving portion sizes of everyday Japanese meals.”; US—“You are a United States registered dietitian. You are deeply familiar with standard single-serving portion sizes of everyday American meals.” The two nationalities were chosen to match the two datasets’ culinary origins, so that persona and food origin could be either aligned or crossed. The contrast of interest is directional: *none → JP* tests whether a Japanese-dietitian frame shifts the estimate, and *none → US* provides a same-structure control that isolates whether any effect is specific to the JP frame rather than a generic consequence of adding a persona. All three clauses were matched in length and register (unified persona text; full wording in Appendix A).

**Factor 2—Portion specification (L0/L1).** Independently of persona, each query fixed the portion cue at one of two levels. *L0* gave no portion information, leaving the model to infer amount from the image alone. *L1* stated that the image showed one standard serving. This factor was included as a reference intervention: L1 supplies, explicitly, the very quantity information that a persona could only affect implicitly, so comparing the JP effect under L0 versus L1 indicates whether a persona acts on the same portion-inference step that an explicit cue addresses.

**Full design.** Crossing the three persona conditions with the two portion levels gives six prompt variants per image; applied across six models and both datasets, this yields 36 model × persona × portion conditions per dataset (Table 3). Persona and portion were varied orthogonally so that their contributions could be read separately.

### 2.4. Metrics

For each nutrient we report the median absolute percentage error, MedAPE = median_i(|ŷ_i − y_i|/y_i × 100), the signed bias in absolute units, bias = mean_i(ŷ_i − y_i) (kcal for energy; grams otherwise), and the mean absolute error, MAE = mean_i(|ŷ_i − y_i|), where ŷ_i and y_i are the predicted and ground-truth values for image i. Percentage errors are computed per image and then aggregated; condition contrasts (e.g., none → JP) are computed on the common set of images with valid predictions under both conditions. We note that percentage-based errors remain unstable for small reference values; the median reduces the influence of extreme observations but does not resolve this denominator problem. For this reason, salt (near-zero references, visually invisible) is excluded from primary claims, absolute-scale metrics are reported alongside, and the shift analyses use image-level paired differences on the absolute scale.

R^2^ is defined as 1 − SS_res/SS_tot, with SS_tot taken around the mean of the observed values and no refitting. Under this definition, R^2^ is not bounded below by zero for an external predictor: negative values indicate that the predictions fit the observations worse than the observed mean, i.e., the predictor carries no usable signal for that nutrient.

Atwater consistency is defined as (4P + 9F + 4C)/E, where P, F, C, and E are the predicted protein, fat, carbohydrate (g), and energy (kcal); values near 1 indicate internally consistent predictions. Observations with ground-truth y ≤ 0 are excluded from percentage-based metrics (one dish, oolong tea, has zero energy).

**Figure 2:**
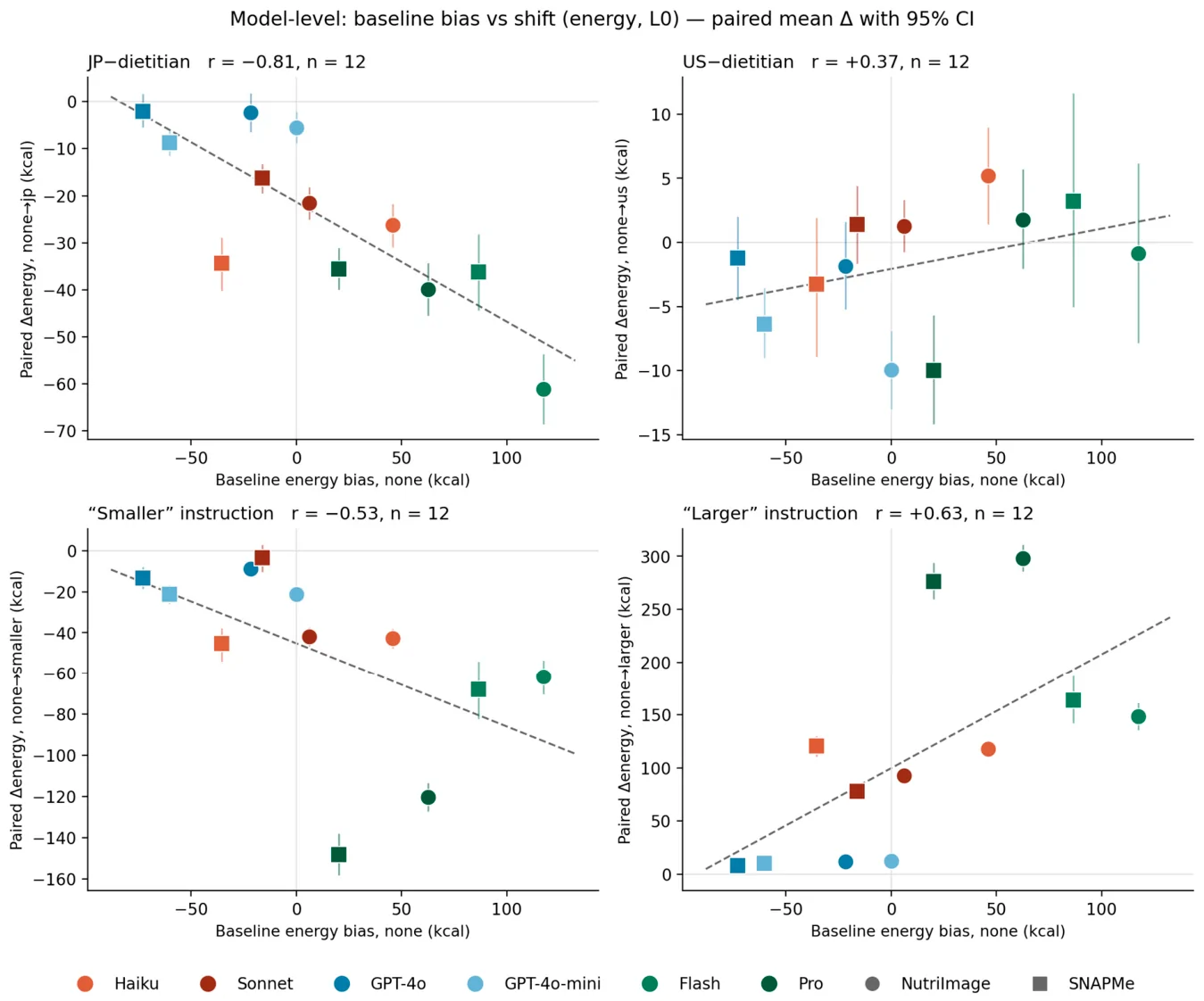
Model-level relationship between baseline energy bias (none condition, L0) and the paired shift under each persona or instruction (mean of image-level paired differences with bootstrap 95% CIs; six models × two datasets; dashed lines: least-squares fits, shown descriptively). The JP-dietitian shift is negative in all twelve combinations and scales with baseline overestimation; the US prompt produces no consistent shift; compliance with the explicit magnitude instructions is vendor-dependent.

**Figure 3:**
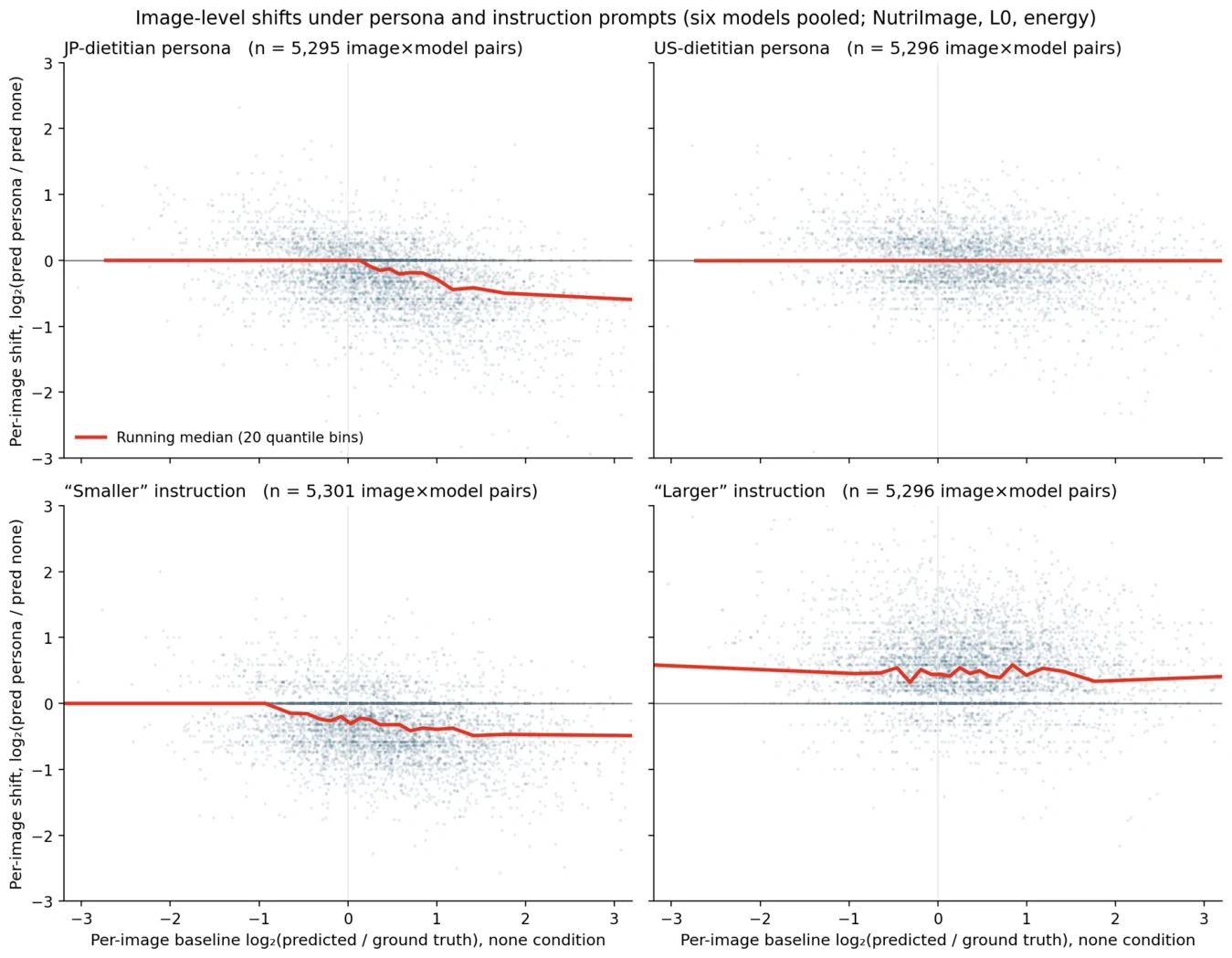
Image-level shifts pooled over the six models (NutriImage, L0, energy). Each point is one image × model pair: the horizontal axis is the per-image baseline log_2_(predicted/ground truth) under none, the vertical axis the per-image shift log_2_(persona/none), and the red line the running median over 20 quantile bins. The JP shift behaves as a conditional correction (near zero for under-estimated images, growing with overestimation); the US prompt serves as an empirical negative control; the explicit instructions produce approximately constant offsets. Per-model median curves are shown in Supplementary Figure S2.

For paired persona contrasts, we report, as the primary statistic, the proportion of images shifted downward, with bootstrap 95% confidence intervals over paired image resampling (2000 resamples), together with the mean and median paired differences (bootstrap CIs), the fraction of exactly zero changes, and paired Cohen’s dz. The downward proportion is primary because several models emit coarsely quantized estimates, producing zero-spikes (up to 58% of images unchanged in some conditions) that render the median uninformative; the zero fraction is reported alongside. Interaction structure is tested with a linear model on image-level paired log-ratios log(ŷ_persona/ŷ_none) with persona × dataset × portion fixed effects and model as a fixed factor, using image-clustered robust standard errors. Equivalence claims on SNAPMe are tested with two one-sided tests (TOST) on ΔMedAPE with a ±5-percentage-point margin (bootstrap 90% CI within the margin; Table S3). Baseline residual comparisons (Table 2) use, per image, the residual averaged over the six models (one independent observation per image), with one-sample *t*-tests against zero, Welch *t*-tests for the between-dataset contrast, Holm correction across the 15 tests, and rank-based tests (Wilcoxon signed-rank, Mann–Whitney) as sensitivity checks, with divergences noted in the table caption. For the model-level relationship between baseline error and persona shift (Figure 2), baseline bias is the signed mean energy error under the none condition (L0), computed per model × dataset on all images with valid predictions, and the persona shift is the mean image-level paired difference defined above; the Pearson correlation over the twelve model × dataset points is reported as a descriptive summary without inferential claims, as the points are not independent. Image-level shift plots (Figure 3) use, per image, the baseline log_2_ ratio of predicted to ground-truth energy on the horizontal axis and the log_2_ ratio of the persona prediction to the none prediction on the vertical axis, summarized by running medians over 20 quantile bins; because both axes share the none prediction, a weak negative association is expected under the null, and the US condition serves as an empirical negative control for this coupling.

### 2.5. Reproducibility

Model endpoints were: claude-haiku-4-5-20251001 and claude-sonnet-4-6 (Anthropic); gpt-4o and gpt-4o-mini (OpenAI); gemini-2.5-flash and gemini-2.5-pro (Google). Main experimental series were executed in late June–early July 2026 (SNAPMe 28 June; NutriImage cropped series 4–5 July); the Flash NutriImage series was re-executed in full on 15–16 August 2026 after an audit found 558/5394 null responses in the July run under API quota pressure (the manuscript reports the re-executed data; per-condition null counts for all series are given in Supplementary Table S4). Sampling parameters were vendor defaults; temperature was not set. Each image × condition was queried once in the main experiments; output stochasticity is quantified separately in Section 3.3 (repeated calls). Images were submitted as JPEG; images exceeding 4,718,592 bytes (4.5 MiB) as base64 were downscaled (area ratio, JPEG quality 85 → 70 → 55 → 40) for the Anthropic and OpenAI APIs and were sent unmodified to the Gemini API. Transient API errors were retried up to six times with exponential backoff; responses failing all retries were recorded as missing.

Model outputs were parsed as JSON after stripping code fences; responses in non-strict JSON (unquoted keys, or missing braces) were rescued by direct regular-expression extraction of the five numeric fields and are flagged in the released prediction files; a response was classified as non-numeric only if any of the five fields could not be extracted as a number. Records with non-numeric outputs were excluded pairwise per contrast. Evaluation sets: NutriImage, 899 of 910 ground-truth records had a corresponding image (11 lacked one), of which 887 with linked ground truth entered the quantitative analyses (sample-size flow diagram in Figure S1); SNAPMe, 1272 of 1478 aggregated non-packaged meals (restricted to photographs classified as containing no legible text). All per-image predictions, ground truth, prompts, and parsing and analysis code are available (Data Availability Statement). All analyses were performed in Python 3.12.3 using NumPy 2.4.4, pandas 3.0.2, SciPy 1.17.1, and statsmodels 0.14.6; figures were produced with Matplotlib 3.10.8.

### 2.6. Unified Analysis Across Models and Datasets

The analyses above characterize persona effects per model and per dataset. To test the central claims free of both factors, we added a unified analysis in which model identity and image source are canceled by design. Pooling the two datasets is supported by their profiles—both consist of ordinary everyday foods with overlapping ground-truth nutrient ranges (Table 1; per-category and per-occasion nutrient profiles in Supplementary Table S7)—and, because every effect is a within-image paired difference, any remaining dataset-level differences in baseline levels cancel by construction. All 2159 images (887 NutriImage, 1272 SNAPMe) were pooled into a single image group, and each image was randomly assigned to exactly one of the six models (balanced assignment, stratified by dataset, fixed random seed). For each condition, the persona effect of an image is the paired difference between the condition and the baseline prediction, computed within the same image and its assigned model; baseline accuracy differences between models therefore cancel by construction, and the 2159 observations are mutually independent. Because API calls are stateless and mutually independent, and the assignment was fixed prior to analysis, this procedure is statistically equivalent to a prospective design in which each image is submitted to one randomly selected model. Mean paired shifts were tested against zero with paired *t*-tests (Wilcoxon signed-rank as a sensitivity check), with Holm correction across all 35 condition–nutrient cells. Because the complete factorial data were retained, the entire analysis can be replayed under independent re-assignments: every effect reported as robust below remained significant in at least 95% of B = 1000 independent random re-assignments. B was chosen so that the Monte Carlo standard error of the reported share is below one percentage point at the 95% threshold. Accuracy preservation was additionally tested by equivalence: for each condition–nutrient cell, the change in pooled MedAPE relative to baseline was computed on a fixed random assignment with bootstrap 90% confidence intervals over image resampling (2000 resamples) against a ±5-percentage-point margin (TOST, with non-inferiority assessed against the +5-point bound), and the point estimates were confirmed stable across 300 independent re-assignments.

## 3. Results

### 3.1. Persona Shifts the Portion Prior Downward (Switch Axis)

Across all six models and both datasets, the JP persona shifted predicted energy downward relative to the none baseline (bias, none → jp, all negative). The US persona produced only small, sign-inconsistent changes, i.e., the effect was asymmetric rather than a generic consequence of adding any persona. On NutriImage (energy, L0), the none → jp bias shift ranged from −2.4 (GPT-4o) to −62.5 (Flash); on SNAPMe, from −2.0 (GPT-4o) to −36.9 (Flash) (Figure 2).

**Figure 4:**
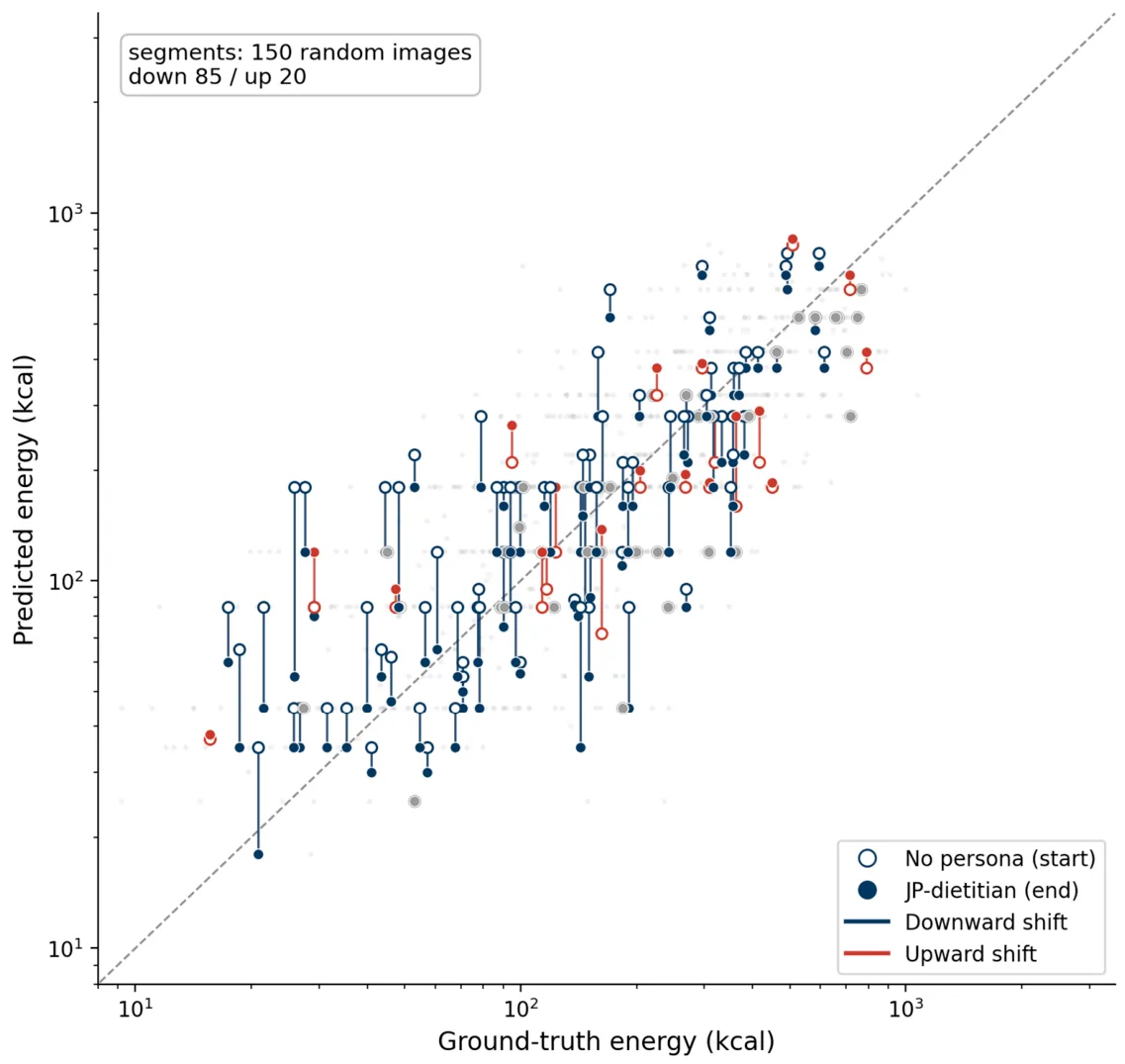
Per-image none → JP shifts for the representative model (Claude Sonnet, NutriImage, L0). For 150 randomly selected images (seed-documented), each vertical segment connects the prediction under none (open circle) to the prediction under the JP prompt (filled circle); blue segments shift downward (*n* = 85), red upward (*n* = 20). Gray points show all evaluated images under none.

Two further properties characterize the switch. First, its depth scaled with the model’s baseline error: across the twelve model × dataset combinations, models that over-estimated more under the none condition shifted further downward under the JP prompt (Pearson r = −0.82; Figure 2; reported descriptively, as the twelve points are not independent—each model contributes one per dataset), and the shift remained negative even where the baseline bias was negative, indicating that the switch fires irrespective of the sign of the baseline error. Second, the same structure is visible at the image level (Figure 3): the median per-image shift was near zero for images the model already under-estimated and grew with per-image overestimation, a pattern absent under the US prompt. Even for GPT-4o, whose mean shift was small and not individually significant (Table 4), the direction was consistent: among images that changed, downward moves significantly outnumbered upward moves on both datasets (sign test, *p* = 0.002 and *p* = 0.032). Figure 4 shows the per-image shifts directly for the representative model, with downward segments outnumbering upward roughly four to one (85 versus 20 of 150 sampled images).

### 3.2. Prompt-Control Conditions

The JP and US prompts co-vary nationality, professional identity, and serving-size framing. To decompose the effect, five control prompts were evaluated (L0, all six models, both full datasets): explicit magnitude instructions with no persona (“You tend to assume smaller [larger] single-serving portion sizes than usual when estimating meals.”); a registered dietitian with no national or cultural specialization (neutral_dietitian) and a Japanese/American homemaker familiar with everyday serving sizes but without nutrition training (jp_home, us_home).

**Figure 5:**
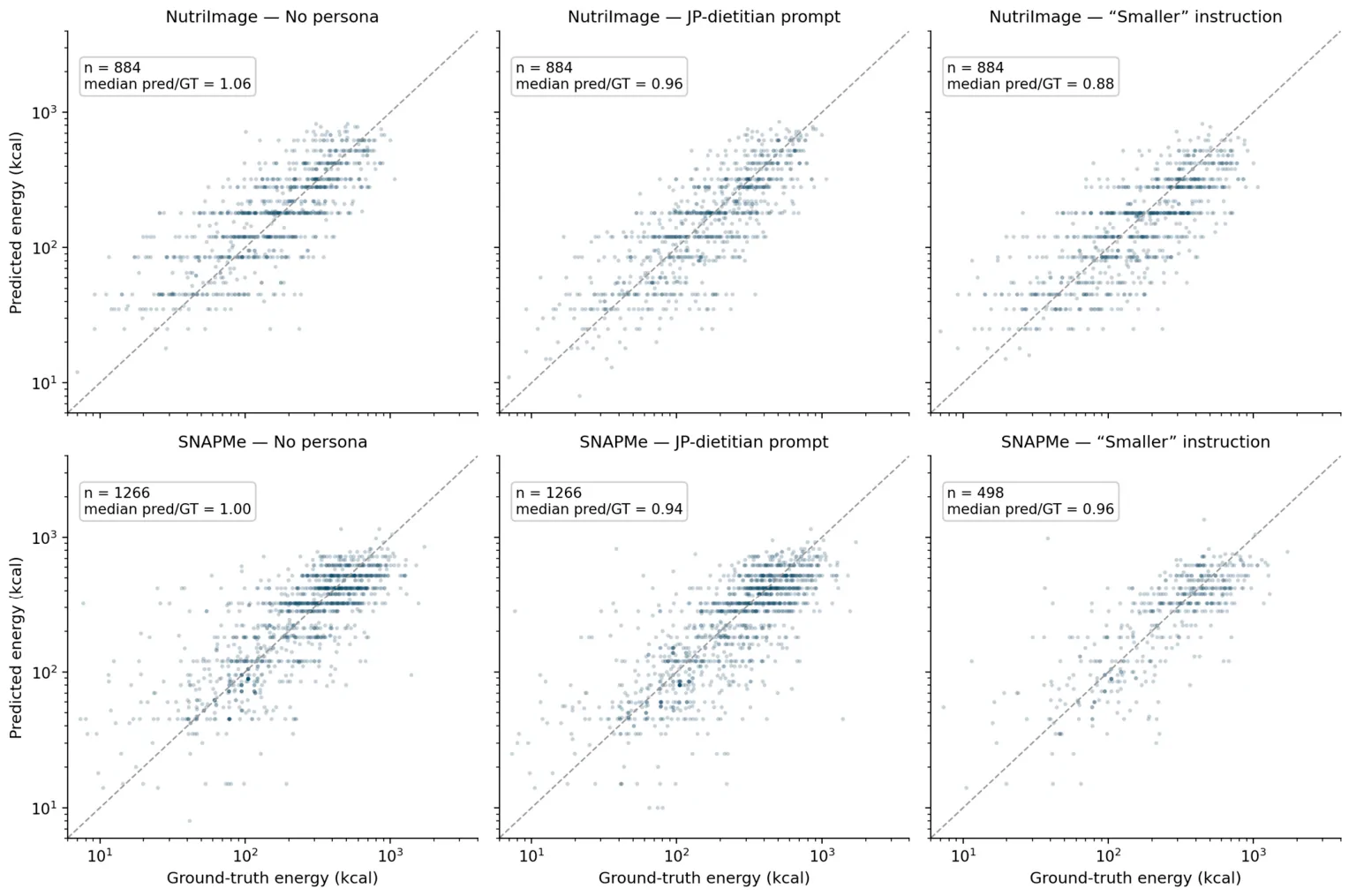
Predicted versus ground-truth energy (log–log) for the representative model (Claude Sonnet) under none, the JP-dietitian prompt, and the smaller instruction, on both datasets. The JP prompt moves the point cloud downward while retaining its shape (median predicted/ground-truth ratio 1.11 → 1.00 on NutriImage), whereas the smaller instruction disperses the cloud and overshoots (0.92). Per-model, per-nutrient versions for all six models are provided in Supplementary Figures S3 and S4.

Both explicit magnitude instructions moved estimates in the instructed direction on average, but compliance was strongly vendor-dependent: the larger instruction shifted the vast majority of images upward for the Anthropic and Gemini models (proportion shifted upward 0.83–1.00), while both OpenAI models responded only weakly in either direction (0.34–0.39 upward under larger; Figure 2, Table S2). Against this backdrop, the JP-dietitian prompt differed from the smaller instruction in kind rather than in degree. At the image level (Figure 3), the JP shift behaved as a conditional correction—near zero for images the model already under-estimated and growing with per-image overestimation—whereas both instructions produced approximately constant offsets irrespective of the baseline, with magnitudes that varied idiosyncratically across models (e.g., mean smaller shifts from −8.8 kcal for GPT-4o to −120.3 kcal for Gemini Pro on the same image set). On NutriImage, the JP prompt moved the median predicted-to-ground-truth ratio from 1.11 to 1.00 for the representative model, whereas the smaller instruction overshot to 0.92 (Figure 5). The JP effect is therefore not reproduced by a paraphrased downscaling command.

The decomposition conditions implicate the nationality/locale axis as the primary contributor to the effect. The Japanese-homemaker prompt (nationality and everyday serving-size familiarity, without nutrition training) reproduced the majority of the JP-dietitian shift in every model (mean paired shifts −1.8 to −49.1 kcal, versus −2.3 to −61.1 kcal under JP), whereas the neutral-dietitian prompt (profession without nationality) produced only small and sign-inconsistent shifts (−11.2 to +6.2 kcal), and both US controls (us, us_home) produced essentially no consistent shift. Professional identity alone is thus not sufficient, and nationality without professional identity is largely sufficient, for the downward shift (Table S2).

### 3.3. Output Stochasticity and Repeated Calls

To separate the persona effect from stochastic output variation, a stratified subsample of 50 NutriImage images (five energy strata × 10; 49 evaluable under the final ground truth) was queried three times under {none, JP} × L0 with two representative models. Sonnet returned identical outputs across repetitions for the majority of images (median within-image SD = 0 kcal), indicating effectively deterministic behavior at default settings; Flash showed genuine stochasticity (median within-image SD = 40.4 kcal). In both models, the JP shift computed on repetition-averaged paired differences was clearly separated from within-condition variability (Sonnet: −25.5 kcal, 95% CI [−35.7, −15.3]; Flash: −81.3 kcal, 95% CI [−108.1, −54.4]; downward proportions 0.69 (34/49) and 0.84 (41/49)). The persona effect cannot be attributed to stochastic output variation. To locate GPT-4o on this spectrum, the same repeated-call design (50 stratified images × 3 repetitions × {none, JP}) was additionally run for GPT-4o: its repetition noise was intermediate (median within-image SD 28.9 kcal), and its repetition-averaged JP shift was small (−11.6 kcal, 95% CI [−22.2, −1.0]; *n* = 48 after one persistent API error; 31/48 downward, Wilcoxon *p* = 0.025)—smaller than its own per-image repetition noise and detectable only after averaging, consistent with GPT-4o’s weak persona response.

### 3.4. Accuracy Consequences Depend on the Dataset’s True Portion Scale (Accuracy Axis)

The same downward switch had opposite accuracy consequences in the two datasets (baseline residuals in Table 2). On NutriImage (small true portions), JP reduced energy MedAPE in all six models (none → jp, L0): Haiku −7.1, Sonnet −2.9, GPT-4o −5.1, GPT-4o-mini −3.6, Flash −19.9, Pro −11.2 pt. On SNAPMe (larger true portions), the same shift did not improve accuracy (improvement essentially absent except for Flash). The vendor ordering was preserved (OpenAI smallest, Anthropic intermediate, Google largest) (Figure 5, Table S1).

### 3.5. Generalization Across Nutrients

**Figure A1:**
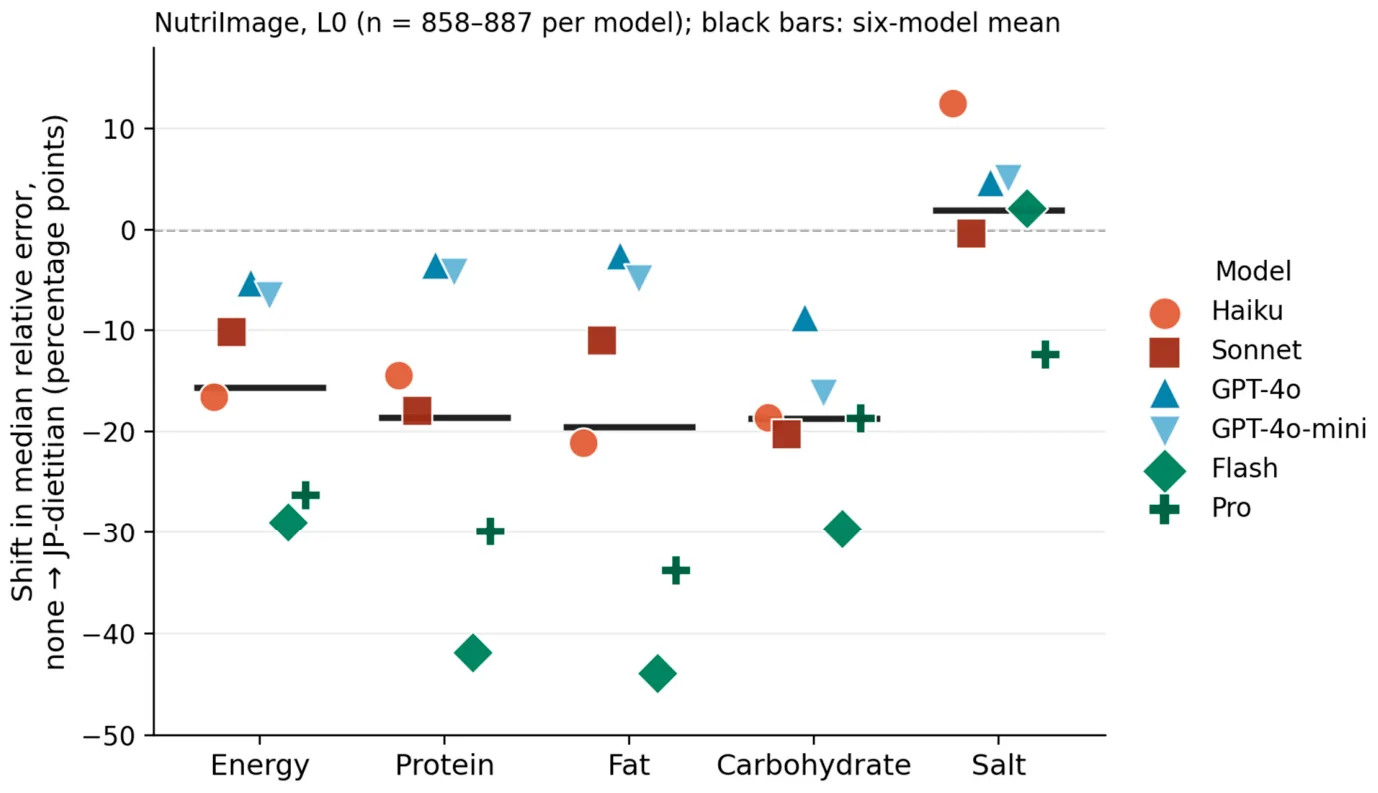
Nutrient-wise shift in the median relative error (none → JP, NutriImage, L0) for the six models; black bars: six-model means. Energy, protein, fat, and carbohydrate shift together (six-model means −15.6 to −19.5 percentage points), while salt scatters around zero without a consistent direction.

**Figure A2:**
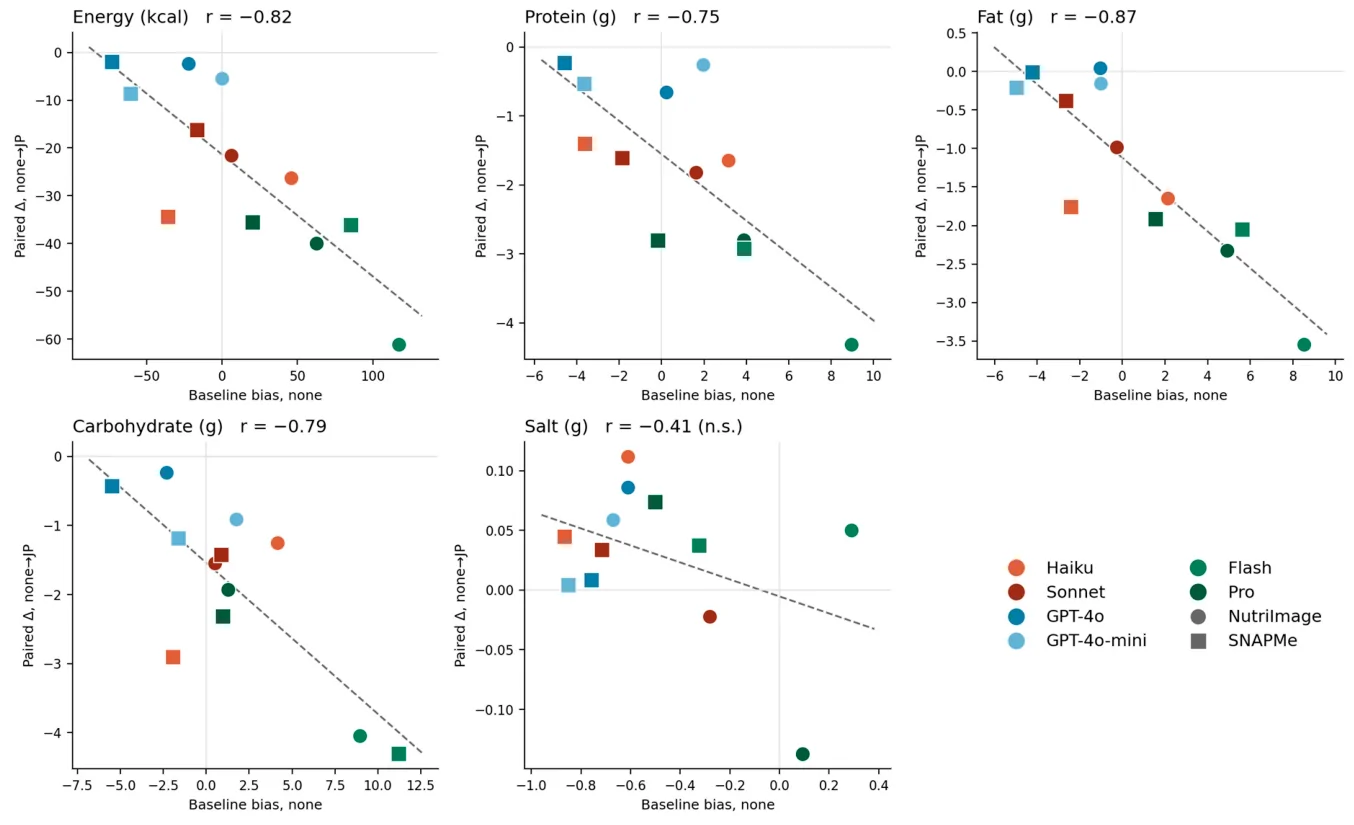
Baseline bias versus paired JP shift, per nutrient (six models × two datasets; dashed lines: least-squares fits, descriptive). The scaling observed for energy holds for the four caloric nutrients, while salt shows no significant relationship.

The JP shift was not confined to energy. On NutriImage, MedAPE improved under none → jp (L0) for energy, protein, and carbohydrate alike (mean −8.3, −11.4, and −12.3 pt, respectively), consistent with a single downward adjustment of estimated amount propagating across the amount-driven nutrients rather than a nutrient-specific effect. Fat also improved on average (−11.1 pt), but its estimates were less stable and are interpreted with caution: unlike portion mass or visibly discrete protein and carbohydrate sources, fat is largely contributed by seasoning, dressing, and cooking oil absorbed into the dish, and is therefore not readily legible from a single image. Salt was excluded from the primary claim: for the Japanese dishes, its estimates were effectively uninformative (R^2^ ≤ 0.16 in all models under all personas, six-model mean 0.075 under JP), reflecting that salt content is essentially invisible in food photographs. On SNAPMe, the downward switch again fired across all nutrients (negative bias shifts) while MedAPE remained essentially unchanged (|ΔMedAPE| ≤ 0.7 pt for every nutrient), reproducing the switch/accuracy separation at the nutrient level (Table 5). The per-model pattern is shown in Figure A1 (Appendix A): the four caloric nutrients shift together in every model, while salt scatters around zero without a consistent direction. The scaling of the shift with baseline bias also held nutrient-by-nutrient for the four caloric nutrients, whereas salt alone showed no significant relationship (Figure A2, Appendix A). Consistent with a common rescaling of amount, the predicted macronutrient energy shares and internal Atwater consistency were essentially unchanged under JP in the two models verified per image, Claude Sonnet and GPT-4o (share changes ≤ 0.9 percentage points; Atwater ratio 0.982–1.000; Table A2).

### 3.6. Robustness to Removing Scene Context (Crop)

To test whether the JP effect depends on the model reading dish or background cues, we re-estimated the original images after cropping to the dish bounding box (same ids, same ground truth). Across the four models available for this comparison, the JP accuracy effect (ΔMedAPE) was negative in 15 of the 16 model × portion × crop cells; the single exception (GPT-4o-mini, L0, original images, +1.7 pt) reversed to −3.1 pt on the cropped images (Appendix A Table A1).

### 3.7. Persona Effects Robust Across Models and Datasets

**Figure 6:**
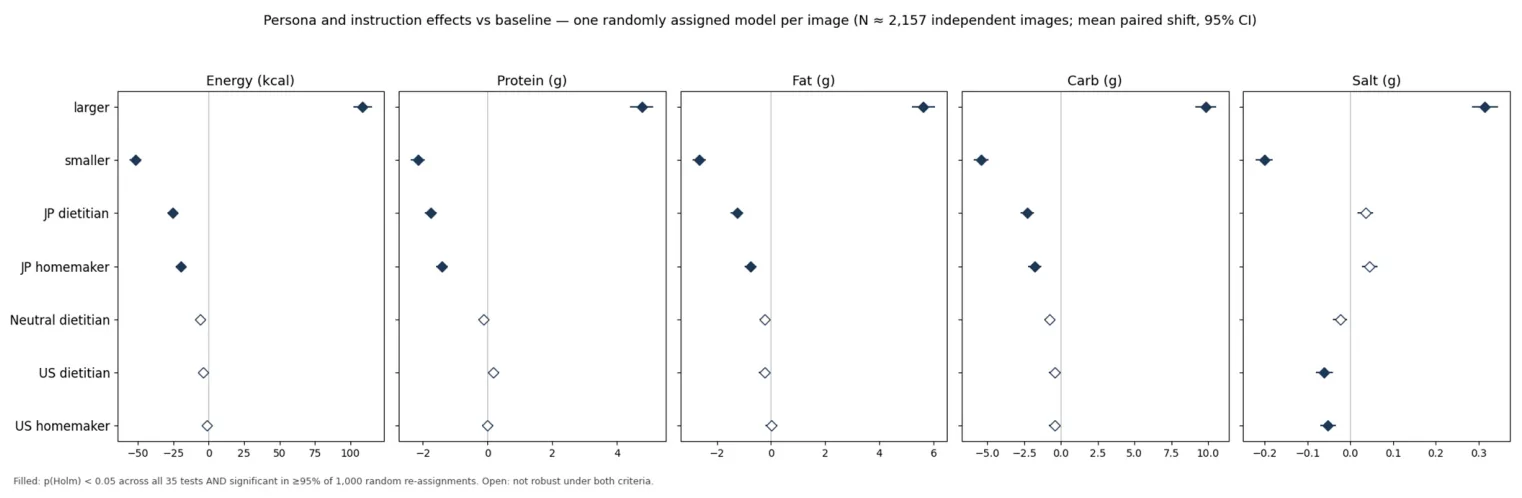
Persona and instruction effects versus baseline under the randomized model-assignment design (*n* ≈ 2157 independent images; mean paired shift with 95% CI). Filled: Holm-corrected *p* < 0.05 and significant in ≥95% of 1000 random re-assignments; open: not robust under both criteria.

Figure 6 summarizes all 35 condition–nutrient effects under the unified analysis. The pattern established in Section 3.1, Section 3.2, Section 3.3, Section 3.4 and Section 3.5 survives the removal of model and dataset identity in full. Both portion instructions moved every nutrient in the instructed direction (energy −51.7 kcal [95% CI −55.9, −47.6] under “smaller”; +108.6 kcal [+102.0, +115.2] under “larger”; all ten cells robust). Both Japanese personas reduced all four macronutrients (energy −25.4 kcal [−28.8, −22.0] for the dietitian and −19.6 kcal [−23.0, −16.2] for the homemaker; protein, fat, and carbohydrate all robust). The US personas moved no macronutrient robustly, yet both reduced salt equivalent (−0.061 g [−0.080, −0.041] and −0.052 g [−0.070, −0.033])—salt being the only nutrient with a robust US-persona effect, in the direction of deepening the systematic under-estimation of salt common to all conditions. The neutral dietitian produced no robust effect in any nutrient (its largest effect, energy −6.0 kcal, was significant on the reported assignment after Holm correction but reproduced in only 20% of re-assignments). Every effect labeled robust was significant in at least 99% of the 1000 re-assignments (100% for every cell except the two US-salt cells, 99.5% and 99.2%). Full statistics for all 35 cells are given in Supplementary Table S5.

The conditional character of the JP correction (Section 3.2, Figure 3) also reproduces in this framework: across deciles of per-image baseline bias, the running median of the jp shift was zero in the five lowest deciles and deepened monotonically to −0.44 log_2_ in the highest (over-estimated stratum *p* = 1 × 10^−86^; under/accurate stratum median 0.00), whereas “smaller” lowered even under-estimated images (median −0.10, *p* = 1 × 10^−34^; paired jp-versus-smaller contrast *p* = 7 × 10^−16^) and “larger” applied a near-constant offset (+0.32 to +0.45 log_2_ in every decile). The median predicted-to-ground-truth ratio moved from 1.084 (baseline) to 0.994 under the JP dietitian and 0.892 under “smaller.”

**Figure 7:**
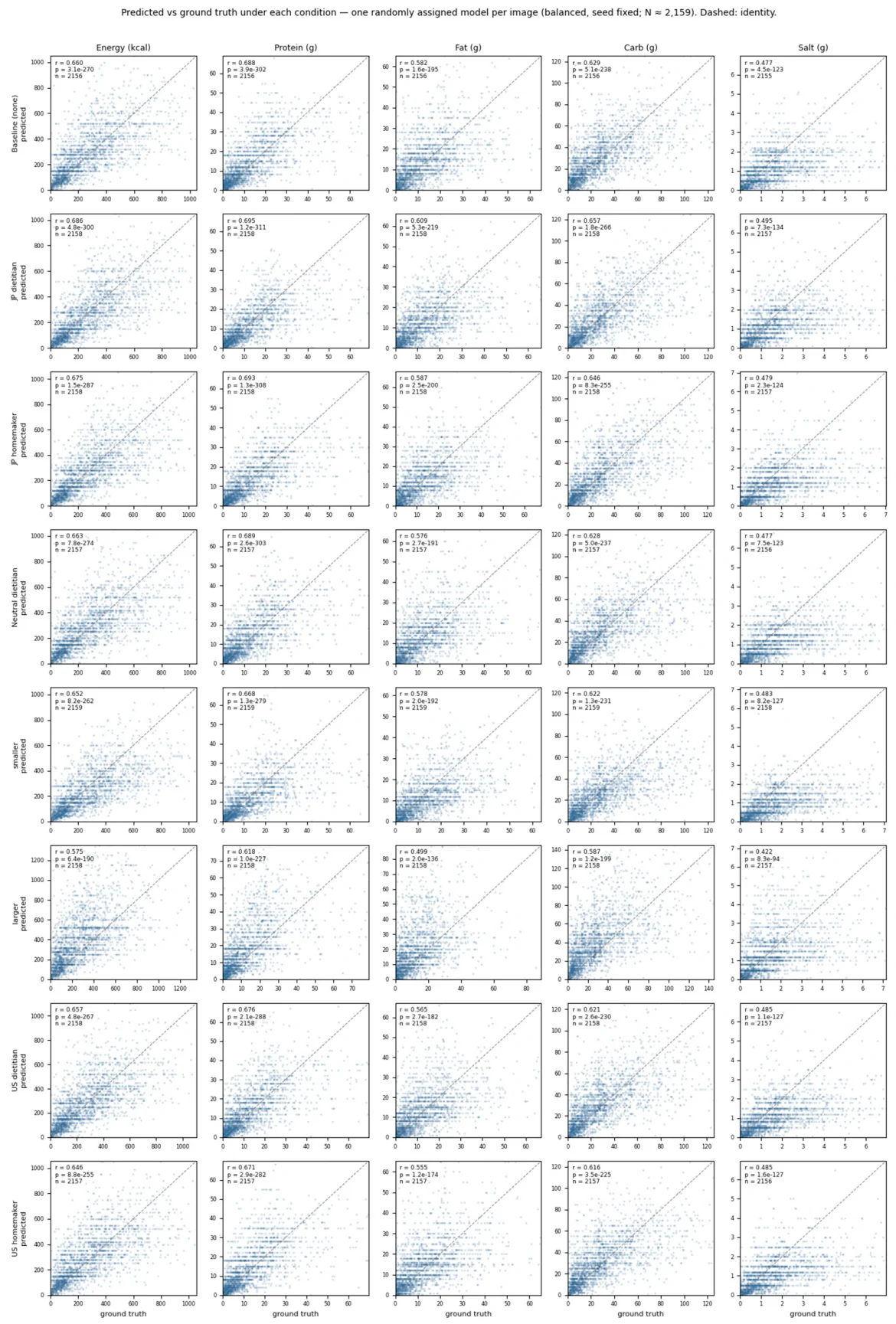
Predicted versus ground-truth values under each condition (one randomly assigned model per image; *n* ≈ 2157 per panel; dashed: identity; axes clipped at the 99.5th percentile for display). Annotations: Pearson r, *p*, *n*.

The image-level structure behind these effects is shown in Figure 7, which plots predicted against ground-truth values for every condition–nutrient pair in the pooled randomized-assignment sample. Under every persona, the point cloud retains its shape and its alignment with the identity line: the personas translate the estimates without degrading their ordering against ground truth (energy r = 0.646–0.686 vs. 0.660 at baseline), and only the “larger” instruction visibly lifts and disperses the cloud (r = 0.575; MAE +55%). The salt panels show the cloud lying below the identity line in every condition, the systematic under-estimation of salt being a property of the task rather than of any persona. Because at *n* ≈ 2157 every correlation in Figure 7 is significant at astronomically small *p* (all *p* < 10^−93^) regardless of condition, the panel *p*-values carry no comparative information and are shown for completeness; inference in this section rests on the Holm-corrected paired effect sizes of Figure 6, and the per-panel correlations, tabulated in Supplementary Table S6, are read as descriptive evidence that the accuracy structure is preserved. This preservation also passes a formal equivalence test: the bootstrap 90% CI of the change in pooled MedAPE relative to baseline lay entirely below the +5-percentage-point degradation bound in all 25 persona-nutrient cells (non-inferiority), and within the full ±5-point equivalence margin in 22 of them; the three exceptions (JP dietitian: energy, protein, carbohydrate) crossed the margin only on the side of improvement. The explicit “smaller” instruction also stayed within the margin in every nutrient, whereas “larger” failed non-inferiority for all four caloric nutrients (ΔMedAPE +18.7 to +20.6 points; cell-level values in Supplementary Table S8). In the same framework, the dispersion of the residuals is likewise preserved: the SD of the pooled energy residual was 174–186 kcal under every persona versus 181 kcal at baseline, and only “larger” inflated it (247 kcal)—the personas move the location of the residual distribution without altering its spread.

In sum, the persona effects reported in this paper are properties of the persona instructions themselves, not of any particular model, vendor, or image source.

## 4. Discussion

The pattern of results is consistent with the JP-dietitian prompt acting on the estimated quantity: the shift is approximately uniform in relative terms across energy, protein, and carbohydrate (−15 to −20 percentage points, six-model average; Figure A1), preserves predicted macronutrient shares (Table A2), and disappears under none of the conditions we tested. We emphasize, however, that no analysis here measures a latent portion-inference stage directly; by “portion prior” we mean, operationally, the distribution of quantity estimates a model produces for a food image in the absence of explicit quantity information—a statistical regularity acquired from training data, not a claimed internal module. Stable macronutrient ratios could also partly reflect models deriving energy internally from their own macronutrient estimates, so Atwater consistency is reported as a consistency check rather than independent proof of quantity rescaling.

The control conditions sharpen what the effect is not. It is not a paraphrased downscaling command: the explicit “smaller” instruction shifted estimates further on average but far less consistently, leaving 26–42% of images unchanged (Table S2), whereas the “larger” instruction was followed almost uniformly by the Anthropic and Gemini models; compliance with explicit magnitude instructions was itself vendor-dependent, with both OpenAI models largely insensitive in either direction (Figure 2), so the persona–instruction contrast cannot be reduced to a single direction-specific asymmetry. The JP prompt attains higher downward consistency than the explicit instruction itself, at a more moderate magnitude, suggesting it operates by changing the reference frame of the estimate rather than by issuing a command against which the model partially resists (Figure 3). The decomposition conditions point in the same direction: a Japanese-homemaker persona without nutrition training reproduced most of the JP shift in every model, whereas a dietitian persona without nationality produced only small shifts, implicating the nationality/locale component—rather than professional identity—as the primary contributor (Section 3.2).

The pattern has a close parallel in human dietary assessment. Trained dietetics students identify foods from meal images far more reliably than they quantify them (79.5% correct identification versus 38% of portion estimates within ±10% of true weight) [11], and the perception of portion size from photographs has long been known to depend on the reference portions the assessor carries [12]. Portion estimation, in other words, is prior-dependent in humans as well; what the persona manipulation appears to control is which population of reference portions the model draws on—the analog, for a VLM, of the culturally learned serving sizes that human assessors bring to the same task.

Two axes must be kept separate, and the SNAPMe result forces the separation. The JP persona lowered predicted portions in every model and both datasets—including SNAPMe—while US produced only small, sign-inconsistent changes; the switch therefore fired identically on US photographs, and only its accuracy consequence differed. On NutriImage (small true portions, over-estimated at baseline), the shift corrected the bias and lowered error; on SNAPMe (larger true portions), the identical shift did not improve accuracy (Figure 5). SNAPMe is thus a *boundary condition* rather than a null result: switching occurs without benefiting accuracy. This bears directly on the report that persona effects are largely inconsistent on objective tasks [9]; part of that inconsistency is expected once the directional switch and its accuracy consequence are distinguished, because the same directional operation helps or does not help depending on the sign of the baseline error. The condition-level scatter plots make this separation visible: every persona leaves the predicted–ground-truth cloud aligned with the identity line, so the switch operates as a translation of the estimates, and its accuracy consequence follows from where the baseline cloud sits relative to that line (Figure 7).

The persona × portion interaction is significant (χ^2^ = 23.1, *p* = 1.5 × 10^−6^): the JP shift attenuates under L1 but does not vanish. The phrase “one standard serving” is culturally and semantically underdetermined; the residual effect may therefore operate through the interpretation of the serving concept itself rather than through an anchoring-like override of explicit quantity information. The present design cannot distinguish whether the persona acts on perception or on inference, and we treat the mechanism as unresolved. Gram-specified servings, visible size references, and culturally neutral serving descriptions are natural next experiments. Whether salt—visually near-invisible yet clinically central to the Japanese diet—could instead be estimated through contextual cues, by linking dish identification to typical seasoning profiles (e.g., miso soup, pickled vegetables), is likewise left to future work. The difficulty is not specific to VLMs: in human dietary assessment, salt intake resists both self-report and visual estimation, with 24 h urinary excretion regarded as the only reliable measure [13].

One possible account is that this behavior reflects the geographic localization of information in web-scale training corpora: everyday quantitative norms such as typical portion sizes are documented largely within their own linguistic and regional communities, and a persona prompt may select which locale’s regularities the model draws upon. Because a food photograph carries no absolute scale, such locale-dependent priors are precisely what a model must fall back on when completing a portion estimate from an image. The asymmetric pattern of Figure 6 is consistent with this account: the Japanese personas robustly moved all four caloric nutrients, the neutral dietitian moved none, and the US personas robustly moved only salt equivalent—the one nutrient that cannot be grounded visually at all, and therefore the one for which the estimate must lean entirely on prior knowledge. This would be consistent with the near-null effect of the US persona (the unmarked default in predominantly English-language corpora) and with the persistence of the effect under the locale-dependent phrase “one standard serving,” although alternative accounts—most notably response calibration introduced during post-training alignment—cannot be excluded with the present data. Direct evidence for such locale selection exists at the level of cultural values: large language models’ default responses align most closely with English-speaking and Protestant-European countries, and “cultural prompting”—instructing the model to answer as a person from a given country—shifts responses toward that country’s survey values [14]. Our results are consistent with the same mechanism operating on a quantitative, visually grounded estimate rather than on expressed values, with the US persona’s near-null macronutrient effect again marking the default. We are pursuing this question in ongoing work.

We note that the SNAPMe photographs retain a fiducial marker, so an absolute size cue was in principle available; the persistence of the JP shift on SNAPMe therefore suggests that the persona effect does not depend on the absence of scale cues, although whether current VLMs actually exploit such markers was not tested here. Complementarily, the JP accuracy effect survived removal of dish-external scene context by cropping to the dish bounding box (Section 3.6, Table A1).

Our central observation—a persona effect that is directional and reproducible across six models—contrasts with the framing of persona effects as largely random on objective tasks [9] and aligns instead with evidence that persona assignment can induce systematic, socio-demographically patterned shifts in model behavior [15]. The distinction from that line of work is the target: prior studies—including evidence in high-profile venues that identity-conditioned models reproduce human-like social identity biases [16]—document persona-induced shifts in social judgment or reasoning [6,15,16], whereas here the shift is on a *quantitative* estimate. Mechanistically, the effect resembles the anchoring biases documented for numerical tasks [7,8], but is triggered by a social label rather than an explicit numeric anchor. The consistent sign across three vendors, with magnitude ordered OpenAI < Anthropic < Google on both axes, we report as an internal observation without attributing it to a specific cause.

The practical reading of these results is narrower than a general accuracy improvement. In real dietary assessment, the per-photo error direction is unknown precisely because ground truth is absent, and a systematically downward prompt can help in one domain and harm in another; our own datasets illustrate both outcomes. The JP prompt is therefore best regarded as a candidate domain-specific calibration intervention, to be adopted only after validation on a representative labeled sample of the target domain—never as a default.

This study originated as an exploratory observation: working with ground truth calculated by Japanese dietitians, we asked how the same estimators would view US meal photographs, observed an unexpected downward shift under the JP prompt, and then symmetrized the design with a US persona. Cross-national differences in typical served portions are documented realities, so the association the models have learned is not an arbitrary stereotype; nevertheless, applying group-level regularities to individual estimates carries the usual risks of ecological fallacy and of reinforcing normative assumptions about national eating habits. This study characterizes a mechanism; it does not recommend deploying nationality labels as calibration devices.

## 5. Limitations

Several limitations apply. First, the primary claim centers on energy; protein and carbohydrate move consistently with it, but fat estimates are less stable (fat is largely contributed by seasoning, dressing, and absorbed cooking oil and is not readily legible from a single image), and salt was excluded as effectively uninformative (visually near-invisible, particularly for the Japanese dishes; bias pinned with R^2^ ≈ 0). Second, the study covers two datasets; the switch/accuracy relationship should be confirmed on further portion regimes before generalizing. Third, a residual JP effect persists even under L1 (explicit one-serving cue), indicating the persona does not act solely on the portion-inference step. Fourth, we document a directional effect consistent with a downward shift of the portion prior; we do not directly measure the prior itself, so the mechanism remains an interpretation rather than a demonstrated quantity. Fifth, the crop-robustness comparison was limited to the four non-Gemini models, as the Gemini models were temporarily subject to API rate limits during that phase; they were nonetheless fully evaluated on the final cropped set, and this comparison can be extended to Gemini if required.

Additional limitations apply: (i) nationality is not isolated from professional identity and serving-size framing in the JP/US prompts—the control conditions decompose but do not fully factorize these components, because a persona requires a concrete person-image and a “nationality-only” condition without a professional or household frame is not constructible, and only two nationality personas were evaluated; (ii) the two datasets differ on multiple dimensions beyond portion scale (cuisine, framing, scene context, meal composition, tableware, fiducial markers), so the dataset-dependent accuracy consequences cannot be attributed to portion scale alone; (iii) commercial model versions and output stochasticity constrain exact reproducibility, although endpoint identifiers, call dates, and a repeated-call analysis are provided; (iv) composition-share analyses at the image level are reported for a subset of models; (v) the practical utility of persona-based calibration depends on access to a labeled validation sample of the target domain; (vi) the learned association exploited here may encode culturally patterned regularities from training data, and its deployment could reinforce normative assumptions (Section 4); (vii) ground-truth values derive from the Fifth Revised Edition (2000) of the Standard Tables of Food Composition in Japan, current at the time of system construction; some composition values differ from the current Eighth Revised Edition, notably in energy conversion and dietary-fiber treatment; (viii) no calibrated scale of persona “strength” exists: the quantitative magnitudes reported are conditional on the specific wordings evaluated and cannot be read as dose–response measurements, which is why the principal claims are stated qualitatively—a calibrated dose–response design, systematically varying persona wording on a standardized-context dataset with measured ground truth such as Nutrition5k, is left to future work.

## 6. Conclusions

Across the six models and two datasets evaluated, the Japanese-dietitian prompt acted as a qualitatively consistent downward influence on predicted quantities: (i) the shift was downward in every model × dataset combination; (ii) its depth scaled with the model’s baseline overestimation, while firing irrespective of the sign of the baseline error; (iii) it differed in kind from explicit magnitude instructions—behaving as a conditional correction concentrated on over-estimated images, whereas the instructions produced approximately constant, vendor-idiosyncratic offsets, with compliance to explicit instructions itself vendor-dependent; (iv) its carrier was the nationality/locale component of the persona rather than professional identity, a Japanese-homemaker prompt reproducing most of the effect and a nationality-free dietitian prompt almost none of it; and (v) its accuracy consequences were dataset-dependent, reducing error on the small-portion single-dish dataset and leaving the larger-portion meal dataset essentially unchanged. The effect approximately preserved predicted macronutrient composition, with salt as the documented exception. A unified randomized model-assignment analysis showed that these effects were robust across the six evaluated models and both image sources, and under repeated random model reassignment. These are qualitative characterizations of the specific prompts evaluated: the reported magnitudes are conditional on the exact wordings used, no calibrated scale of persona strength exists, and generalization to other nationality or persona prompts remains untested.

## Figures and Tables

**Table 1:** Profiles of the two datasets. Portion-weight rows are computed on the released ground-truth files (*n* = 892 dishes/1478 meals); the remaining quantitative rows on the evaluation sets. NutriImage composition is a heuristic classification from dish names (per-dish mapping provided with the released data; per-category and per-occasion nutrient distributions in Supplementary Table S7); SNAPMe composition is the eating occasion recorded in the benchmark metadata [2].

	NutriImage	SNAPMe
Composition	Protein mains 42%; vegetable sides 25%; staples (rice/noodle/bread) 14%; salads/pickles 7%; desserts/fruit 5%; soups 4%; beverages 3%	Breakfast 33%; dinner 25%; lunch 22%; snacks 16%; drink-only 4% (median 2 food items per meal, IQR 1–4)
Evaluation set	887 dishes	1272 meals
Portion weight, median (IQR), g	130 (81–213)	240 (123–372)
Energy (ground truth), median (IQR), kcal	168 (86–317)	273 (105–453)

**Table 2:** Baseline residuals (predicted − ground truth) under the none condition (L0): per-image residual averaged over the six models (one independent observation per image; NutriImage *n* = 887, SNAPMe *n* = 1272). Mean with 95% CI and one-sample *t*-test *p* (Holm-corrected across the 15 tests in this table), median, and Welch *t*-test *p* for the between-dataset contrast. Rank-based sensitivity tests (Wilcoxon signed-rank, Mann–Whitney) agree except where mean and median diverge: the SNAPMe under-estimation of energy, protein, and fat and the NutriImage salt effect are mean-driven (near-zero medians), and the carbohydrate between-dataset difference is significant only by the rank test.

Nutrient	NutriImage Mean [95% CI] (*p*)	Median	SNAPMe Mean [95% CI] (*p*)	Median	Δ(NI − SN) *p*
Energy (kcal)	+35.1 [27.1, 43.1] (3 × 10^−16^)	+40.7	−12.8 [−22.3, −3.3] (0.024)	+3.8	4 × 10^−13^
Protein (g)	+3.3 [2.9, 3.7] (6 × 10^−58^)	+2.6	−1.6 [−2.2, −1.1] (9 × 10^−8^)	+0.0	2 × 10^−44^
Fat (g)	+2.2 [1.7, 2.7] (3 × 10^−19^)	+1.9	−1.1 [−1.7, −0.5] (0.001)	+0.0	2 × 10^−16^
Carbohydrate (g)	+2.4 [1.2, 3.6] (7 × 10^−4^)	+3.8	+0.9 [−0.2, +2.0] (0.14)	+2.3	0.14
Salt (g)	−0.26 [−0.40, −0.13] (7 × 10^−4^)	+0.01	−0.66 [−0.73, −0.59] (2 × 10^−72^)	−0.19	2 × 10^−6^

**Table 3:** Experimental conditions. All conditions were evaluated for all six models; persona clauses are given verbatim in Section 2.3 and Appendix A. Numbers are the maximum evaluated images per model (per-cell ns vary slightly with pairwise exclusion of non-numeric responses; Supplementary Table S4).

Condition	Role	Portion Levels	NutriImage	SNAPMe
none	Baseline (no persona)	L0, L1	887	1272
jp	Japanese-dietitian persona (primary)	L0, L1	887	1272
us	US-dietitian persona (symmetric control)	L0, L1	887	1272
smaller	Explicit downscaling instruction	L0	887	1272
larger	Explicit upscaling instruction	L0	887	1272
neutral_dietitian	Profession without nationality	L0	887	1272
jp_home	Nationality without profession	L0	887	1272
us_home	US nationality without profession	L0	887	1272

**Table 4:** Image-level paired statistics for the none → JP-dietitian contrast (energy, kcal; L0). CIs by bootstrap over paired image resampling (2000 resamples). P(down): proportion of images shifted downward (primary statistic); P(zero): fraction exactly unchanged; dz: paired Cohen’s dz. Full statistics for all conditions, including L1 and the US and control prompts, are given in Supplementary Table S2.

Model	*n*	Mean Δ [95% CI] (kcal)	Median Δ	P(down) [95% CI]	P(zero)	dz
**NutriImage**						
Claude Haiku	886	−26.3 [−30.5, −22.0]	0	0.43 [0.40, 0.46]	0.44	−0.40
Claude Sonnet	887	−21.6 [−24.8, −18.3]	−10	0.55 [0.52, 0.58]	0.31	−0.44
GPT-4o	882	−2.3 [−6.0, +1.6]	0	0.38 [0.35, 0.42]	0.31	−0.04
GPT-4o-mini	887	−5.5 [−8.6, −2.1]	0	0.35 [0.32, 0.38]	0.44	−0.11
Gemini Flash	887	−61.1 [−68.3, −54.3]	−50	0.74 [0.71, 0.76]	0.04	−0.57
Gemini Pro	887	−39.9 [−45.3, −34.7]	−32	0.74 [0.71, 0.77]	0.03	−0.52
**SNAPMe**						
Claude Haiku	1272	−34.4 [−40.3, −29.2]	0	0.47 [0.44, 0.49]	0.41	−0.34
Claude Sonnet	1272	−16.2 [−19.4, −13.2]	0	0.44 [0.41, 0.46]	0.40	−0.29
GPT-4o	1268	−2.1 [−5.5, +1.2]	0	0.36 [0.33, 0.38]	0.33	−0.03
GPT-4o-mini	1272	−8.7 [−11.3, −5.8]	0	0.37 [0.34, 0.40]	0.44	−0.18
Gemini Flash	1270	−35.8 [−43.8, −28.2]	−20	0.63 [0.60, 0.65]	0.06	−0.26
Gemini Pro	1272	−35.5 [−39.8, −31.3]	−28	0.68 [0.66, 0.71]	0.05	−0.45

**Table 5:** Mean none → JP shift per nutrient across six models (L0): change in MedAPE (percentage points) and change in bias (kcal for energy; g for macronutrients and salt), and JP-condition R^2^, for NutriImage and SNAPMe.

Nutrient	NutriImage dMedAPE	NutriImage dBias	NutriImage JP R2	SNAPMe dMedAPE	SNAPMe dBias	SNAPMe JP R2
Energy	−8.3	−26.3	+0.48	−0.1	−22.3	+0.41
Protein	−11.4	−1.9	+0.36	−0.7	−1.6	+0.50
Carbohydrate	−12.3	−1.7	+0.51	0.6	−2.1	+0.28
Fat	−11.1	−1.5	+0.26	−0.2	−1.1	+0.31
Salt	−2.1	0.0	+0.07	0.2	0.0	+0.28

## Data Availability

Restrictions apply to the availability of part of the data. The NutriImage dataset analyzed in this study is archived on Zenodo (Concept DOI: 10.5281/zenodo.21944881; version 1.1, used in this study: 10.5281/zenodo.21961032, accessed on 30 August 2026). Within that record, the dietitian-calculated ground-truth CSV and a README documenting provenance are openly available, whereas the 887 text-removed images are available under restricted access for academic research via the repository’s access-request function; redistribution is not permitted. Third-party data were also analyzed in this study. The SNAPMe dataset is openly available in Ag Data Commons at https://doi.org/10.15482/USDA.ADC/1528346 (accessed on 30 August 2026), and is described in Larke et al. [2]. The data generated in this study—per-image model predictions for all evaluated model–condition–dataset combinations (132 result files), the ground-truth linkage tables for both datasets, the exact prompt texts for all conditions, and the parsing and analysis code—are available in the Supplementary Material (reproducibility_package.zip; file inventory in the enclosed README).

## Supplementary Materials

The following supporting information can be downloaded at: https://www.mdpi.com/article/10.3390/nu18172892/s1, Table S1. Complete metrics; Table S2. Image-level paired statistics for all conditions (energy, kcal); Table S3. Equivalence testing on SNAPMe (TOST, ±5-percentage-point margin); Table S4. Valid predictions per condition; Table S5. Unified randomized model-assignment analysis: all 35 condition-nutrient effects; Table S6. Pearson correlations with ground truth under the randomized model-assignment design; Table S7. Ground-truth distributions and composition profiles of the two datasets (complements main-text Table 1 and Table 2); Table S8. Equivalence (TOST) of pooled MedAPE under each condition (randomized-assignment sample); Figure S1. Sample-size flow; Figure S2. Per-model median shift curves; Figure S3. Per-model, per-nutrient predicted versus ground truth (NutriImage); Figure S4. Per-model, per-nutrient predicted versus ground truth (SNAPMe).

## Appendix A

The full per-condition results are provided as Supplementary Materials; the complete 4-metric × 5-nutrient × 36-condition table for both datasets is provided as Supplementary Table S1 (full_metrics). The exact prompt wording used in all conditions is reproduced verbatim below.

### Appendix A.1. Base Task Instruction (All Conditions)

Look at this food image. Estimate the nutritional content based solely on visual appearance—color, shape, ingredients, and portion size. Do NOT read any text, labels, or numbers visible in the image. Use only visual information. Provide a brief one-line explanation of your reasoning. Respond ONLY in JSON format: {energy_kcal: value, protein_g: value, fat_g: value, carb_g: value, salt_g: value, basis: one-line reasoning}.

### Appendix A.2. Persona Clauses (Prepended as the System Prompt)

**none:** (no persona; empty system prompt).

**JP:** You are a Japanese registered dietitian (kanri-eiyoshi). You are deeply familiar with standard single-serving portion sizes of everyday Japanese meals.

**US:** You are a United States registered dietitian. You are deeply familiar with standard single-serving portion sizes of everyday American meals.

### Appendix A.3. Portion Clause (Appended to the Base Instruction)

**L0:** (no portion clause).

**L1:** The image shows exactly one standard single serving for one person (ichinin-ikkai-bun). Estimate the nutritional content of that single serving.

**Table A1:** Crop robustness: JP effect on energy MedAPE (none → JP, percentage points) for original vs. cropped images, matched by id on the released ground truth (*n* = 682–690 per condition).

Model	Portion	JP Effect Original (pp)	JP Effect Cropped (pp)	*n*	GT Mismatch
Haiku	L0	−5.6	−8.2	685	0
Haiku	L1	−4.6	−7.6	688	0
Sonnet	L0	−0.1	−3.3	689	0
Sonnet	L1	−0.2	−2.2	690	0
GPT-4o	L0	−3.5	−4.3	683	0
GPT-4o	L1	−3.0	−1.9	682	0
GPT-4o-mini	L0	+1.7	−3.1	690	0
GPT-4o-mini	L1	−2.9	−4.8	690	0

**Table A2:** Composition neutrality (none vs. JP, L0): predicted macronutrient energy shares and internal Atwater consistency, verified per image for Sonnet and GPT-4o.

Model	*n*	Condition	P Share (%)	F Share (%)	C Share (%)	Atwater (4P + 9F + 4C)/E
Claude Sonnet 4.6	660	None	19.0	33.3	44.0	0.986
Claude Sonnet 4.6	660	JP	18.4	34.2	43.6	0.982
GPT-4o	596	None	16.9	37.5	43.7	1.000
GPT-4o	596	JP	16.7	37.5	43.6	1.000

## References

[1] Nakagawa S., Yamamoto A. (2026). Prompt Engineering and Model Selection for LLM-Based Nutritional Estimation from Food Images: A Multi-Dataset Investigation. Nutrients.

[2] Larke J.A., Chin E.L., Bouzid Y.Y., Nguyen T., Vainberg Y., Lee D.H., Pirsiavash H., Smilowitz J.T., Lemay D.G. (2023). Surveying Nutrient Assessment with Photographs of Meals (SNAPMe): A Benchmark Dataset of Food Photos for Dietary Assessment. Nutrients.

[3] Fridolfsson J., Sjöberg E., Thiwång M., Pettersson S. (2025). Performance Evaluation of 3 Large Language Models for Nutritional Content Estimation from Food Images. Curr. Dev. Nutr..

[4] O’Hara C., Kent G., Flynn A.C., Gibney E.R., Timon C.M. (2025). An Evaluation of ChatGPT for Nutrient Content Estimation from Meal Photographs. Nutrients.

[5] Kamruzzaman M., Kim G.L. (2025). Exploring Changes in Nation Perception with Nationality-Assigned Personas in LLMs. Proceedings of the 2025 Conference on Empirical Methods in Natural Language Processing (EMNLP 2025).

[6] Kamruzzaman M., Shovon M.M.I., Kim G.L. (2024). Investigating Subtler Biases in LLMs: Ageism, Beauty, Institutional, and Nationality Bias in Generative Models. Findings of the Association for Computational Linguistics: ACL 2024.

[7] Tversky A., Kahneman D. (1974). Judgment under Uncertainty: Heuristics and Biases. Science.

[8] Lou J., Sun Y. (2026). Anchoring Bias in Large Language Models: An Experimental Study. J. Comput. Soc. Sci..

[9] Zheng M., Pei J., Logeswaran L., Lee M., Jurgens D. (2024). When “A Helpful Assistant” Is Not Really Helpful: Personas in System Prompts Do Not Improve Performances of Large Language Models. Findings of the Association for Computational Linguistics: EMNLP 2024.

[10] Thames Q., Karpur A., Norris W., Xia F., Panait L., Weyand T., Sim J. (2021). Nutrition5k: Towards Automatic Nutritional Understanding of Generic Food. Proceedings of the IEEE/CVF Conference on Computer Vision and Pattern Recognition (CVPR).

[11] Howes E., Boushey C.J., Kerr D.A., Tomayko E.J., Cluskey M. (2017). Image-Based Dietary Assessment Ability of Dietetics Students and Interns. Nutrients.

[12] Nelson M., Atkinson M., Darbyshire S. (1994). Food Photography I: The Perception of Food Portion Size from Photographs. Br. J. Nutr..

[13] Champagne C.M., Cash K.C. (2013). Assessment of Salt Intake: How Accurate Is It?. Proc. Nutr. Soc..

[14] Tao Y., Viberg O., Baker R.S., Kizilcec R.F. (2024). Cultural Bias and Cultural Alignment of Large Language Models. PNAS Nexus.

[15] Gupta S., Shrivastava V., Deshpande A., Kalyan A., Clark P., Sabharwal A., Khot T. Bias Runs Deep: Implicit Reasoning Biases in Persona-Assigned LLMs. Proceedings of the Twelfth International Conference on Learning Representations (ICLR).

[16] Hu T., Kyrychenko Y., Rathje S., Collier N., van der Linden S., Roozenbeek J. (2025). Generative Language Models Exhibit Social Identity Biases. Nat. Comput. Sci..

