# Supplementary material for: A Japanese-Dietitian Prompt Systematically Shifts Portion Estimates in LLM-Based Nutrient Estimation from Food Images: A Multi-Dataset, Multi-Model Study
Source: Nutrients. 2026 Sep 3;18(17):2892. doi: 10.3390/nu18172892 (PMC13567310; doi:10.3390/nu18172892)
Supplement: Supplementary file 1 [file nutrients-18-02892-s001.zip › Supplementary_Materials_draft.pdf]

Supplementary Materials

A Japanese-Dietitian Prompt Systematically Shifts Portion Estimates in LLM-Based Nutrient Estimation from Food Images: A Multi-Dataset, Multi-Model Study — Nakagawa & Yamamoto

Contents: Table S1 (separate spreadsheet file: TableS1_full_metrics.xlsx); Tables S2–S8; Figures S1–S4.

Table S1. Complete metrics (separate file)

The complete 4-metric (MedAPE, bias, MAE, R^2) × 5-nutrient × 36-condition (6 models × {none, JP, US} × {L0, L1}) table for both datasets is provided as a separate spreadsheet, TableS1_full_metrics.xlsx (360 rows).

Table S2. Image-level paired statistics for all conditions (energy, kcal)

Paired none→condition statistics per dataset × model (energy; bootstrap 95% CIs over 2,000 paired image resamples; P(down) = proportion of images shifted downward with bootstrap CI; P(zero) = fraction exactly unchanged; dz = paired Cohen's dz). L0 rows cover all seven conditions; L1 rows cover the JP and US personas.

dataset	model	portion	contrast	n	meanΔ (kcal)	95% CI	medianΔ	P(down)	P(down) lo	P(down) hi	P(zero)	dz
NI	4o	L0	none->jp	882	-2.3	[-6.0, 1.6]	0.0	0.3832	0.3503	0.415	0.3141	-0.039
NI	4o	L0	none->us	880	-1.87	[-5.4, 1.6]	0.0	0.3352	0.3034	0.3682	0.3659	-0.036
NI	4o	L0	none->smaller	883	-8.76	[-12.2, -5.4]	0.0	0.3805	0.3499	0.4122	0.3499	-0.161
NI	4o	L0	none->larger	878	12.18	[8.2, 16.2]	0.0	0.2517	0.2244	0.2813	0.3576	0.204
NI	4o	L0	none->neutral_dietitian	873	6.21	[2.8, 10.	0.0	0.2749	0.244	0.3047	0.3631	0.116

dataset	model	portion	contrast	n	meanΔ (kcal)	95% CI	medianΔ	P(down)	P(down) lo	P(down) hi	P(zero)	dz
						0]						
NI	4o	L0	none->jp_home	878	-3.12	[-7.1, 0.7]	0.0	0.3804	0.3485	0.4123	0.3212	-0.053
NI	4o	L0	none->us_home	881	0.54	[-3.0, 4.1]	0.0	0.3156	0.2838	0.3462	0.3632	0.01
NI	4o	L1	none->jp	881	-7.23	[-10.9, -3.6]	0.0	0.4211	0.3882	0.4529	0.2894	-0.129
NI	4o	L1	none->us	886	-4.5	[-7.8, -1.1]	0.0	0.3691	0.3386	0.4029	0.3397	-0.083
NI	4o-mini	L0	none->jp	887	-5.46	[-8.6, -2.1]	0.0	0.3484	0.3179	0.3788	0.4431	-0.112
NI	4o-mini	L0	none->us	887	-9.94	[-13.1, -6.9]	0.0	0.3348	0.3044	0.3653	0.4769	-0.214
NI	4o-mini	L0	none->smaller	887	-21.19	[-24.3, -18.1]	0.0	0.4521	0.4194	0.4848	0.4183	-0.448
NI	4o-mini	L0	none->larger	887	12.56	[9.7, 15.7]	0.0	0.1838	0.1578	0.2097	0.4758	0.271
NI	4o-mini	L0	none->neutral_dietitian	887	-2.84	[-5.6, 0.1]	0.0	0.2762	0.2469	0.3078	0.5085	-0.065
NI	4o-mini	L0	none->jp_home	887	-1.81	[-4.9, 1.4]	0.0	0.3044	0.274	0.3371	0.4679	-0.039

dataset	model	portion	contrast	n	meanΔ (kcal)	95% CI	medianΔ	P(down)	P(down) lo	P(down) hi	P(zero)	dz
NI	4o-mini	L0	none->us_home	887	-3.61	[-6.5, -0.6]	0.0	0.2773	0.2469	0.3067	0.4972	-0.082
NI	4o-mini	L1	none->jp	887	-1.91	[-5.0, 1.2]	0.0	0.3698	0.3393	0.4014	0.3991	-0.04
NI	4o-mini	L1	none->us	887	-4.46	[-7.2, -1.5]	0.0	0.3269	0.2965	0.3563	0.4464	-0.104
NI	flash	L0	none->jp	887	-61.14	[-68.3, -54.3]	-50.0	0.7362	0.708	0.7644	0.0417	-0.57
NI	flash	L0	none->us	887	-0.83	[-7.8, 6.5]	0.0	0.478	0.4453	0.5096	0.0947	-0.008
NI	flash	L0	none->smaller	887	-61.57	[-69.3, -53.9]	-50.0	0.7486	0.7182	0.7768	0.0519	-0.527
NI	flash	L0	none->larger	887	148.59	[136.7, 160.9]	110.0	0.1342	0.1105	0.1567	0.0316	0.812
NI	flash	L0	none->neutral_dietitian	887	-5.63	[-12.5, 1.4]	0.0	0.4837	0.451	0.5163	0.106	-0.053
NI	flash	L0	none->jp_home	887	-49.05	[-55.7, -42.1]	-40.0	0.699	0.6685	0.7294	0.0643	-0.481
NI	flash	L0	none->us_home	887	6.86	[0.1,	0.0	0.425	0.3923	0.4566	0.115	0.066

dataset	model	portion	contrast	n	meanΔ (kcal)	95% CI	medianΔ	P(down)	P(down) lo	P(down) hi	P(zero)	dz
						14.0]						
NI	flash	L1	none->jp	887	-44.91	[-51.8, -38.1]	-40.0	0.6922	0.6629	0.7227	0.0564	-0.42
NI	flash	L1	none->us	886	5.35	[-1.2, 11.8]	0.0	0.447	0.4142	0.4797	0.0892	0.054
NI	haiku	L0	none->jp	886	-26.26	[-30.5, -22.0]	0.0	0.4278	0.3962	0.4594	0.4391	-0.395
NI	haiku	L0	none->us	886	5.19	[1.7, 8.8]	0.0	0.1704	0.1456	0.1941	0.5327	0.093
NI	haiku	L0	none->smaller	886	-42.83	[-47.4, -38.7]	-30.0	0.5271	0.4932	0.5598	0.4153	-0.651
NI	haiku	L0	none->larger	886	117.89	[112.6, 123.3]	100.0	0.0045	0.0011	0.009	0.1174	1.4
NI	haiku	L0	none->neutral_dietitian	886	-11.23	[-14.6, -7.6]	0.0	0.2652	0.237	0.2957	0.5869	-0.213
NI	haiku	L0	none->jp_home	886	-31.85	[-36.1, -27.9]	0.0	0.447	0.4142	0.4786	0.4537	-0.504
NI	haiku	L0	none->us_home	886	2.89	[-1.1,	0.0	0.1806	0.1569	0.2065	0.5564	0.05

dataset	model	portion	contrast	n	meanΔ (kcal)	95% CI	medianΔ	P(down)	P(down) lo	P(down) hi	P(zero)	dz
						6.7]						
NI	haiku	L1	none->jp	887	-18.06	[-21.7, -14.3]	0.0	0.3946	0.3641	0.4262	0.4611	-0.317
NI	haiku	L1	none->us	887	7.52	[4.1, 10.8]	0.0	0.1714	0.1477	0.1973	0.5254	0.144
NI	pro	L0	none->jp	887	-39.9	[-45.3, -34.7]	-32.0	0.7362	0.708	0.7666	0.0349	-0.515
NI	pro	L0	none->us	887	1.76	[-2.1, 5.8]	0.0	0.451	0.4194	0.4825	0.1048	0.03
NI	pro	L0	none->smaller	887	-120.29	[-126.9, -114.2]	-90.0	0.9786	0.9684	0.9876	0.0101	-1.207
NI	pro	L0	none->larger	887	298.09	[287.2, 309.4]	280.0	0.0	0.0	0.0	0.0	1.771
NI	pro	L0	none->neutral_dietitian	887	-6.96	[-10.8, -3.1]	-5.0	0.5265	0.4938	0.5581	0.0992	-0.12
NI	pro	L0	none->jp_home	887	-30.75	[-35.3, -26.2]	-25.0	0.7046	0.6719	0.7339	0.0586	-0.44
NI	pro	L0	none->us_home	887	0.86	[-3.3, 5.1]	0.0	0.4386	0.4047	0.4724	0.1026	0.013

dataset	model	portion	contrast	n	meanΔ (kcal)	95% CI	medianΔ	P(down)	P(down) lo	P(down) hi	P(zero)	dz
NI	pro	L1	none->jp	887	-32.12	[-36.6, -27.3]	-23.0	0.7091	0.6787	0.7384	0.0406	-0.461
NI	pro	L1	none->us	887	3.35	[-0.5, 7.2]	0.0	0.4521	0.4205	0.4837	0.0699	0.058
NI	sonnet	L0	none->jp	887	-21.59	[-24.8, -18.3]	-10.0	0.5513	0.5197	0.5829	0.3089	-0.438
NI	sonnet	L0	none->us	887	1.25	[-0.7, 3.3]	0.0	0.177	0.1522	0.2029	0.6009	0.042
NI	sonnet	L0	none->smaller	887	-41.8	[-45.9, -37.8]	-30.0	0.6697	0.6381	0.7001	0.2638	-0.674
NI	sonnet	L0	none->larger	887	93.03	[89.4, 97.1]	100.0	0.0045	0.0011	0.009	0.0507	1.583
NI	sonnet	L0	none->neutral_dietitian	887	-4.37	[-6.7, -2.3]	0.0	0.2503	0.2232	0.2785	0.6156	-0.135
NI	sonnet	L0	none->jp_home	887	-17.35	[-20.3, -14.5]	0.0	0.478	0.4431	0.5096	0.398	-0.388
NI	sonnet	L0	none->us_home	887	2.62	[-0.0, 5.6]	0.0	0.2063	0.1804	0.2334	0.566	0.065
NI	sonnet	L1	none->jp	887	-12.	[-15.	0.0	0.4634	0.4284	0.4972	0.3698	-0.2

dataset	model	portion	contrast	n	meanΔ (kcal)	95% CI	medianΔ	P(down)	P(down) lo	P(down) hi	P(zero)	dz
					75	9, -9.8]						81
NI	sonnet	L1	none->us	887	1.01	[-1.1, 3.1]	0.0	0.2018	0.177	0.2289	0.5603	0.032
SN	4o	L0	none->jp	1272	-2.15	[-5.6, 1.2]	0.0	0.3577	0.3318	0.3836	0.3302	-0.035
SN	4o	L0	none->us	1272	-1.11	[-4.2, 1.9]	0.0	0.3333	0.3074	0.3593	0.3443	-0.019
SN	4o	L0	none->smaller	1272	-12.86	[-16.1, -9.5]	0.0	0.4198	0.3915	0.4465	0.32	-0.215
SN	4o	L0	none->larger	1272	11.47	[7.7, 15.2]	0.0	0.2602	0.2374	0.2854	0.3412	0.171
SN	4o	L0	none->neutral_dietitian	1272	6.32	[3.2, 9.6]	0.0	0.2642	0.2406	0.2893	0.3671	0.106
SN	4o	L0	none->jp_home	1272	-0.98	[-4.8, 2.8]	0.0	0.3381	0.3129	0.3656	0.3428	-0.014
SN	4o	L0	none->us_home	1272	1.11	[-2.6, 4.8]	0.0	0.3145	0.2893	0.3404	0.3664	0.016
SN	4o	L1	none->jp	1271	-1.39	[-4.4, 1.6]	0.0	0.3234	0.2982	0.3509	0.3792	-0.025
SN	4o	L1	none->us	1272	-3.44	[-6.4, -0.7]	0.0	0.3467	0.3208	0.3726	0.3679	-0.064
SN	4o-mini	L0	none->jp	1272	-8.66	[-11.4, -	0.0	0.3703	0.3451	0.397	0.4403	-0.175

dataset	model	portion	contrast	n	meanΔ (kcal)	95% CI	medianΔ	P(down)	P(down) lo	P(down) hi	P(zero)	dz
						5.9]						
SN	4o-mini	L0	none->us	1272	-6.35	[-9.0, -3.6]	0.0	0.3388	0.3137	0.3632	0.4568	-0.131
SN	4o-mini	L0	none->smaller	1272	-22.1	[-24.8, -19.4]	0.0	0.445	0.4182	0.4701	0.4481	-0.46
SN	4o-mini	L0	none->larger	1272	13.43	[10.4, 16.3]	0.0	0.2099	0.1879	0.2327	0.4552	0.248
SN	4o-mini	L0	none->neutral_dietitian	1272	-1.85	[-4.6, 0.8]	0.0	0.2909	0.2649	0.3153	0.4898	-0.039
SN	4o-mini	L0	none->jp_home	1272	-4.64	[-7.2, -1.8]	0.0	0.3294	0.305	0.3569	0.4607	-0.093
SN	4o-mini	L0	none->us_home	1272	-3.18	[-5.8, -0.7]	0.0	0.3121	0.2877	0.3373	0.4764	-0.067
SN	4o-mini	L1	none->jp	1271	2.38	[-0.2, 4.9]	0.0	0.2628	0.2392	0.2872	0.491	0.051
SN	4o-mini	L1	none->us	1271	-1.2	[-3.7, 1.2]	0.0	0.273	0.2486	0.299	0.5098	-0.028
SN	flash	L0	none->jp	1270	-35.79	[-43.8, -28.2]	-20.0	0.6268	0.6	0.6528	0.0598	-0.259
SN	flash	L0	none->us	1270	1.96	[-6.0, 10.	0.0	0.4882	0.4598	0.5142	0.1008	0.013

dataset	model	portion	contrast	n	meanΔ (kcal)	95% CI	medianΔ	P(down)	P(down) lo	P(down) hi	P(zero)	dz
						2]						
SN	flash	L0	none->smaller	1270	-70.47	[-78.6, -62.2]	-45.0	0.7165	0.6929	0.7417	0.063	-0.469
SN	flash	L0	none->larger	1268	151.61	[139.5, 164.1]	100.0	0.1664	0.1459	0.1885	0.0331	0.677
SN	flash	L0	none->neutral_dietitian	1270	3.66	[-3.7, 11.0]	0.0	0.4882	0.4606	0.5165	0.1118	0.027
SN	flash	L0	none->jp_home	1267	-25.43	[-33.1, -18.1]	-10.0	0.5627	0.5343	0.5896	0.0852	-0.185
SN	flash	L0	none->us_home	1270	-2.87	[-9.6, 4.2]	0.0	0.4472	0.4197	0.4748	0.1094	-0.022
SN	flash	L1	none->jp	1226	-24.71	[-31.3, -18.1]	-19.0	0.6044	0.5767	0.6313	0.0457	-0.212
SN	flash	L1	none->us	1219	5.58	[-1.5, 12.8]	0.0	0.4282	0.4003	0.4553	0.1124	0.044
SN	haiku	L0	none->jp	1272	-34.38	[-40.4, -29.0]	0.0	0.4662	0.4395	0.4945	0.408	-0.339
SN	haiku	L0	none->us	1272	-3.24	[-8.6, 1.7]	0.0	0.2241	0.2013	0.2469	0.4898	-0.034

dataset	model	portion	contrast	n	meanΔ (kcal)	95% CI	medianΔ	P(down)	P(down) lo	P(down) hi	P(zero)	dz
SN	haiku	L0	none->smaller	1272	-47.45	[-52.9, -42.2]	-17.5	0.5322	0.5055	0.5597	0.386	-0.483
SN	haiku	L0	none->larger	1272	114.56	[108.8, 120.2]	100.0	0.0102	0.0047	0.0157	0.1439	1.078
SN	haiku	L0	none->neutral_dietitian	1272	-9.19	[-13.7, -4.9]	0.0	0.2335	0.2107	0.2563	0.5998	-0.114
SN	haiku	L0	none->jp_home	1272	-28.47	[-34.0, -23.6]	0.0	0.3813	0.3553	0.4072	0.4866	-0.299
SN	haiku	L0	none->us_home	1271	0.28	[-4.2, 4.2]	0.0	0.1912	0.1699	0.2124	0.5445	0.004
SN	haiku	L1	none->jp	1272	-17.46	[-20.7, -14.2]	0.0	0.423	0.3947	0.4505	0.4363	-0.298
SN	haiku	L1	none->us	1272	6.05	[2.9, 9.2]	0.0	0.173	0.1525	0.1934	0.5527	0.111
SN	pro	L0	none->jp	1272	-35.53	[-39.9, -31.3]	-28.0	0.6847	0.6588	0.7099	0.0464	-0.454
SN	pro	L0	none->us	1272	-9.99	[-14.4, -5.8]	-5.0	0.5212	0.4929	0.5495	0.1179	-0.131
SN	pro	L0	none-	12	-	[-	-	0.97	0.96	0.98	0.01	-

dataset	model	portion	contrast	n	meanΔ (kcal)	95% CI	medianΔ	P(down)	P(down) lo	P(down) hi	P(zero)	dz
			>smaller	72	151.16	157.7, -144.8]	135.0	41	54	27	97	1.294
SN	pro	L0	none->larger	1272	275.62	[266.5, 285.9]	250.0	0.0039	0.0008	0.0079	0.0055	1.506
SN	pro	L0	none->neutral_dietitian	1272	-10.44	[-13.9, -6.7]	-3.0	0.5149	0.4882	0.5425	0.1321	-0.16
SN	pro	L0	none->jp_home	1272	-25.16	[-29.3, -20.9]	-19.0	0.6242	0.5975	0.6509	0.0739	-0.337
SN	pro	L0	none->us_home	1272	-5.36	[-9.0, -1.6]	-2.0	0.5055	0.478	0.5322	0.1179	-0.079
SN	pro	L1	none->jp	1272	-31.92	[-35.9, -27.8]	-25.0	0.6643	0.6376	0.691	0.0322	-0.436
SN	pro	L1	none->us	1272	-10.54	[-14.2, -6.8]	-5.0	0.5252	0.4984	0.5519	0.1423	-0.157
SN	sonnet	L0	none->jp	1272	-16.22	[-19.2, -13.2]	0.0	0.4371	0.4096	0.4638	0.4009	-0.291
SN	sonnet	L0	none->us	1272	1.39	[-1.4, 4.3]	0.0	0.2154	0.1926	0.2382	0.5582	0.026
SN	son	L0	none-	12	-	[-	0.0	0.34	0.31	0.37	0.37	-

dataset	model	portion	contrast	n	meanΔ (kcal)	95% CI	medianΔ	P(down)	P(down) lo	P(down) hi	P(zero)	dz
	net		>smaller	72	5.26	8.9, -1.8]		51	84	11	11	0.08
SN	sonnet	L0	none->larger	1272	75.57	[71.5, 79.7]	70.0	0.0173	0.0102	0.0244	0.1517	1.045
SN	sonnet	L0	none->neutral_dietitian	1272	-4.44	[-7.4, -1.6]	0.0	0.2445	0.2201	0.2681	0.6195	-0.089
SN	sonnet	L0	none->jp_home	1272	-16.1	[-19.0, -13.2]	0.0	0.4143	0.3884	0.4434	0.4623	-0.3
SN	sonnet	L0	none->us_home	1272	-8.58	[-11.5, -5.7]	0.0	0.2932	0.2689	0.3192	0.5448	-0.165
SN	sonnet	L1	none->jp	1272	-13.88	[-16.4, -11.6]	0.0	0.4363	0.4096	0.4638	0.3994	-0.308
SN	sonnet	L1	none->us	1272	-0.72	[-2.6, 1.3]	0.0	0.2193	0.1965	0.2414	0.5786	-0.02

Table S3. Equivalence testing on SNAPMe (TOST, ±5-percentage-point margin)

Change in MedAPE (none→JP) per model × portion × nutrient on SNAPMe, with bootstrap 90% and 95% CIs; a cell is equivalent when the 90% CI lies within ±5 percentage points.

model	portion	nutrient	n	dMedAPE	ci90_lo	ci90_hi	ci95_lo	ci95_hi	equivalent_pm5pt
haiku	L0	energy_k	126	2.48	0.8	4.11	0.48	4.45	YES

model	portion	nutrient	n	dMedAPE	ci90_lo	ci90_hi	ci95_lo	ci95_hi	equivalent_pm5pt
		cal	9						
haiku	L0	protein_g	1251	0.68	-0.62	3.23	-1.04	3.51	YES
haiku	L0	fat_g	1232	0.98	-1.37	2.97	-1.81	3.4	YES
haiku	L0	carb_g	1241	1.39	-0.62	3.61	-1.02	4.0	YES
haiku	L0	salt_g	1258	-0.03	-2.16	1.31	-2.47	1.76	YES
haiku	L1	energy_kcal	1269	2.35	0.58	3.94	0.29	4.37	YES
haiku	L1	protein_g	1251	2.21	0.73	4.63	0.34	5.01	YES
haiku	L1	fat_g	1232	-1.01	-2.79	1.67	-3.2	2.11	YES
haiku	L1	carb_g	1241	1.09	-0.3	3.62	-0.62	4.18	YES
haiku	L1	salt_g	1258	-0.21	-2.47	1.23	-2.79	1.52	YES
sonnet	L0	energy_kcal	1269	0.47	-0.93	1.85	-1.23	2.14	YES
sonnet	L0	protein_g	1251	0.65	-0.9	2.17	-1.16	2.55	YES
sonnet	L0	fat_g	1232	1.68	-0.95	3.72	-1.48	4.21	YES
sonnet	L0	carb_g	1241	1.95	-0.5	3.41	-0.91	3.74	YES
sonnet	L0	salt_g	1258	2.5	1.28	4.42	1.11	4.81	YES
sonnet	L1	energy_kcal	1269	1.98	0.14	2.97	-0.17	3.24	YES
sonnet	L1	protein_g	1251	1.76	0.26	3.4	0.03	3.85	YES
sonnet	L1	fat_g	1232	3.47	0.86	5.29	0.55	5.7	no
sonnet	L1	carb_g	1241	3.21	0.98	5.05	0.58	5.4	no
sonn	L1	salt_g	125	3.02	1.49	4.92	1.23	5.26	YES

model	portion	nutrient	n	dMedAPE	ci90_lo	ci90_hi	ci95_lo	ci95_hi	equivalent_pm5pt
et			8						
4o	L0	energy_kcal	1265	-0.47	-2.09	0.91	-2.38	1.26	YES
4o	L0	protein_g	1247	-2.11	-3.94	0.0	-4.29	0.29	YES
4o	L0	fat_g	1228	-1.32	-3.14	0.43	-3.51	0.89	YES
4o	L0	carb_g	1237	-2.44	-4.26	-0.82	-4.64	-0.49	YES
4o	L0	salt_g	1254	0.56	-0.72	2.37	-1.07	2.67	YES
4o	L1	energy_kcal	1268	-0.79	-1.89	0.65	-2.09	0.87	YES
4o	L1	protein_g	1250	-0.27	-2.2	1.1	-2.61	1.41	YES
4o	L1	fat_g	1231	-0.42	-2.55	1.17	-2.9	1.7	YES
4o	L1	carb_g	1240	0.52	-0.81	2.04	-1.13	2.32	YES
4o	L1	salt_g	1257	-0.09	-1.92	1.38	-2.22	1.9	YES
4o-mini	L0	energy_kcal	1269	-0.35	-1.5	1.04	-1.72	1.25	YES
4o-mini	L0	protein_g	1251	-0.99	-2.91	0.84	-3.22	1.17	YES
4o-mini	L0	fat_g	1232	-0.36	-2.49	1.63	-2.97	2.0	YES
4o-mini	L0	carb_g	1241	-0.15	-1.92	1.8	-2.3	2.04	YES
4o-mini	L0	salt_g	1258	-1.72	-3.41	-0.22	-3.74	0.0	YES
4o-mini	L1	energy_kcal	1268	-2.59	-3.46	-1.03	-3.67	-0.7	YES
4o-mini	L1	protein_g	1250	-0.23	-2.07	1.43	-2.4	1.75	YES
4o-mini	L1	fat_g	1231	-0.65	-2.44	1.27	-2.76	1.67	YES
4o-	L1	carb_g	124	1.47	-0.77	2.48	-1.28	2.8	YES

model	portion	nutrient	n	dMedAPE	ci90_lo	ci90_hi	ci95_lo	ci95_hi	equivalent_pm5pt
mini			0						
4o-mini	L1	salt_g	1257	-2.17	-3.36	-0.78	-3.59	-0.59	YES
flash	L0	energy_kcal	1205	-4.51	-7.55	-2.03	-8.06	-1.59	no
flash	L0	protein_g	1187	-4.33	-7.34	-1.41	-7.84	-0.91	no
flash	L0	fat_g	1172	-4.49	-8.14	-0.96	-8.95	-0.18	no
flash	L0	carb_g	1178	-2.89	-5.81	0.31	-6.42	0.9	no
flash	L0	salt_g	1195	-0.02	-2.22	2.5	-2.76	2.9	YES
flash	L1	energy_kcal	1223	-0.64	-3.3	0.71	-3.76	1.04	YES
flash	L1	protein_g	1205	-3.42	-6.12	-0.78	-6.71	-0.28	no
flash	L1	fat_g	1189	2.16	-2.09	5.61	-2.7	6.32	no
flash	L1	carb_g	1196	0.14	-2.96	3.25	-3.41	3.98	YES
flash	L1	salt_g	1213	0.02	-2.43	2.56	-2.8	2.97	YES
pro	L0	energy_kcal	1269	1.71	-0.26	3.65	-0.69	4.08	YES
pro	L0	protein_g	1251	2.15	-0.16	4.14	-0.63	4.54	YES
pro	L0	fat_g	1232	2.26	-0.87	4.77	-1.34	5.29	YES
pro	L0	carb_g	1241	5.04	2.73	6.97	2.47	7.46	no
pro	L0	salt_g	1258	0.06	-2.41	2.06	-2.79	2.45	YES
pro	L1	energy_kcal	1269	0.38	-1.3	2.1	-1.66	2.51	YES
pro	L1	protein_g	1251	3.52	1.17	5.38	0.7	5.66	no
pro	L1	fat_g	123	0.43	-2.73	3.65	-3.26	4.25	YES

model	portion	nutrient	n	dMedAPE	ci90_lo	ci90_hi	ci95_lo	ci95_hi	equivalent_pm5pt
			2						
pro	L1	carb_g	1241	5.22	3.09	7.22	2.7	7.52	no
pro	L1	salt_g	1258	0.17	-2.03	2.02	-2.44	2.3	YES

Table S4. Valid predictions per condition

Usable (valid-numeric) predictions per dataset × model × condition × portion; missing = evaluation-set size (887 NutriImage / 1,272 SNAPMe) minus usable. The Gemini Flash NutriImage series reflects the full re-execution of 15–16 August 2026 (Section 2.5).

dataset	model	persona	portion	usable	expected	missing
NI	4o	jp	L0	885	887	2
NI	4o	jp	L1	882	887	5
NI	4o	jp_home	L0	881	887	6
NI	4o	larger	L0	881	887	6
NI	4o	neutral_dietitian	L0	876	887	11
NI	4o	none	L0	884	887	3
NI	4o	none	L1	886	887	1
NI	4o	smaller	L0	886	887	1
NI	4o	us	L0	883	887	4
NI	4o	us	L1	887	887	0
NI	4o	us_home	L0	884	887	3
NI	4o-mini	jp	L0	887	887	0
NI	4o-mini	jp	L1	887	887	0
NI	4o-mini	jp_home	L0	887	887	0
NI	4o-mini	larger	L0	887	887	0
NI	4o-mini	neutral_dietitian	L0	887	887	0
NI	4o-mini	none	L0	887	887	0
NI	4o-mini	none	L1	887	887	0
NI	4o-mini	smaller	L0	887	887	0
NI	4o-mini	us	L0	887	887	0
NI	4o-mini	us	L1	887	887	0
NI	4o-mini	us_home	L0	887	887	0
NI	flash	jp	L0	887	887	0

dataset	model	persona	portion	usable	expected	missing
NI	flash	jp	L1	887	887	0
NI	flash	jp_home	L0	887	887	0
NI	flash	larger	L0	887	887	0
NI	flash	neutral_dietitian	L0	887	887	0
NI	flash	none	L0	887	887	0
NI	flash	none	L1	887	887	0
NI	flash	smaller	L0	887	887	0
NI	flash	us	L0	887	887	0
NI	flash	us	L1	886	887	1
NI	flash	us_home	L0	887	887	0
NI	haiku	jp	L0	887	887	0
NI	haiku	jp	L1	887	887	0
NI	haiku	jp_home	L0	887	887	0
NI	haiku	larger	L0	887	887	0
NI	haiku	neutral_dietitian	L0	886	887	1
NI	haiku	none	L0	886	887	1
NI	haiku	none	L1	887	887	0
NI	haiku	smaller	L0	887	887	0
NI	haiku	us	L0	886	887	1
NI	haiku	us	L1	887	887	0
NI	haiku	us_home	L0	886	887	1
NI	pro	jp	L0	887	887	0
NI	pro	jp	L1	887	887	0
NI	pro	jp_home	L0	887	887	0
NI	pro	larger	L0	887	887	0
NI	pro	neutral_dietitian	L0	887	887	0
NI	pro	none	L0	887	887	0
NI	pro	none	L1	887	887	0
NI	pro	smaller	L0	887	887	0
NI	pro	us	L0	887	887	0
NI	pro	us	L1	887	887	0
NI	pro	us_home	L0	887	887	0
NI	sonnet	jp	L0	887	887	0
NI	sonnet	jp	L1	887	887	0
NI	sonnet	jp_home	L0	887	887	0

dataset	model	persona	portion	usable	expected	missing
NI	sonnet	larger	L0	887	887	0
NI	sonnet	neutral_dietitian	L0	887	887	0
NI	sonnet	none	L0	887	887	0
NI	sonnet	none	L1	887	887	0
NI	sonnet	smaller	L0	887	887	0
NI	sonnet	us	L0	887	887	0
NI	sonnet	us	L1	887	887	0
NI	sonnet	us_home	L0	887	887	0
SN	4o	jp	L0	1272	1272	0
SN	4o	jp	L1	1271	1272	1
SN	4o	jp_home	L0	1272	1272	0
SN	4o	larger	L0	1272	1272	0
SN	4o	neutral_dietitian	L0	1272	1272	0
SN	4o	none	L0	1272	1272	0
SN	4o	none	L1	1272	1272	0
SN	4o	smaller	L0	1272	1272	0
SN	4o	us	L0	1272	1272	0
SN	4o	us	L1	1272	1272	0
SN	4o	us_home	L0	1272	1272	0
SN	4o-mini	jp	L0	1272	1272	0
SN	4o-mini	jp	L1	1272	1272	0
SN	4o-mini	jp_home	L0	1272	1272	0
SN	4o-mini	larger	L0	1272	1272	0
SN	4o-mini	neutral_dietitian	L0	1272	1272	0
SN	4o-mini	none	L0	1272	1272	0
SN	4o-mini	none	L1	1271	1272	1
SN	4o-mini	smaller	L0	1272	1272	0
SN	4o-mini	us	L0	1272	1272	0
SN	4o-mini	us	L1	1272	1272	0
SN	4o-mini	us_home	L0	1272	1272	0
SN	flash	jp	L0	1272	1272	0
SN	flash	jp	L1	1237	1272	35
SN	flash	jp_home	L0	1269	1272	3
SN	flash	larger	L0	1270	1272	2
SN	flash	neutral_dietitian	L0	1272	1272	0

dataset	model	persona	portion	usable	expected	missing
SN	flash	none	L0	1270	1272	2
SN	flash	none	L1	1261	1272	11
SN	flash	smaller	L0	1272	1272	0
SN	flash	us	L0	1272	1272	0
SN	flash	us	L1	1230	1272	42
SN	flash	us_home	L0	1272	1272	0
SN	haiku	jp	L0	1272	1272	0
SN	haiku	jp	L1	1272	1272	0
SN	haiku	jp_home	L0	1272	1272	0
SN	haiku	larger	L0	1272	1272	0
SN	haiku	neutral_dietitian	L0	1272	1272	0
SN	haiku	none	L0	1272	1272	0
SN	haiku	none	L1	1272	1272	0
SN	haiku	smaller	L0	1272	1272	0
SN	haiku	us	L0	1272	1272	0
SN	haiku	us	L1	1272	1272	0
SN	haiku	us_home	L0	1271	1272	1
SN	pro	jp	L0	1272	1272	0
SN	pro	jp	L1	1272	1272	0
SN	pro	jp_home	L0	1272	1272	0
SN	pro	larger	L0	1272	1272	0
SN	pro	neutral_dietitian	L0	1272	1272	0
SN	pro	none	L0	1272	1272	0
SN	pro	none	L1	1272	1272	0
SN	pro	smaller	L0	1272	1272	0
SN	pro	us	L0	1272	1272	0
SN	pro	us	L1	1272	1272	0
SN	pro	us_home	L0	1272	1272	0
SN	sonnet	jp	L0	1272	1272	0
SN	sonnet	jp	L1	1272	1272	0
SN	sonnet	jp_home	L0	1272	1272	0
SN	sonnet	larger	L0	1272	1272	0
SN	sonnet	neutral_dietitian	L0	1272	1272	0
SN	sonnet	none	L0	1272	1272	0
SN	sonnet	none	L1	1272	1272	0

dataset	model	persona	portion	usable	expected	missing
SN	sonnet	smaller	L0	1272	1272	0
SN	sonnet	us	L0	1272	1272	0
SN	sonnet	us	L1	1272	1272	0
SN	sonnet	us_home	L0	1272	1272	0

Table S5. Unified randomized model-assignment analysis: all 35 condition-nutrient effects

Mean paired shift versus baseline per condition-nutrient cell (units: kcal for energy, g otherwise), computed on the fixed random assignment (seed 20260820; assignment procedure in the released analysis code), with t-based 95% CIs, paired t-test p Holm-corrected across all 35 cells, Wilcoxon signed-rank sensitivity p, and the share of 1,000 independent random re-assignments in which the cell remained significant (Holm-corrected within each re-assignment). Robust = Holm-significant on the reported assignment and significant in ≥95% of re-assignments (20 of 35 cells).

Condition	Nutrient	n	Mean shift	95% CI	p (Holm)	p (Wilcoxon)	Share significant (1,000 re-assignments)	Robust
JP dietitian	Energy	2155	-25.4	[-28.8, -22.0]	8.1e-45	2.7e-60	1.000	yes
JP dietitian	Protein	2155	-1.8	[-2.0, -1.6]	2.9e-68	6.6e-81	1.000	yes
JP dietitian	Fat	2155	-1.3	[-1.5, -1.0]	1.2e-25	4.4e-36	1.000	yes
JP dietitian	Carb	2155	-2.3	[-2.7, -1.8]	4.6e-22	1.8e-38	1.000	yes
JP dietitian	Salt	2155	+0.036	[+0.017, +0.054]	0.002	4.8e-04	0.557	no
JP homemaker	Energy	2155	-19.6	[-23.0, -16.2]	6.3e-27	2.3e-42	1.000	yes
JP homemaker	Protein	2155	-1.4	[-1.6, -1.2]	1.5e-46	8.4e-61	1.000	yes
JP	Fat	215	-0.755	[-	9.2e-	4.5e-18	1.000	yes

Condition	Nutrient	n	Mean shift	95% CI	p (Holm)	p (Wilcoxon)	Share significant (1,000 re-assignments)	Robust
homemaker		5		0.980, -0.531]	10			
JP homemaker	Carb	2155	-1.8	[-2.2, -1.3]	9.8e-13	3.2e-25	1.000	yes
JP homemaker	Salt	2155	+0.045	[+0.027, +0.063]	2.0e-05	1.7e-05	0.770	no
Neutral dietitian	Energy	2155	-6.0	[-8.9, -3.2]	4.3e-04	3.3e-07	0.196	no
Neutral dietitian	Protein	2155	-0.137	[-0.289, +0.015]	0.307	0.084	0.009	no
Neutral dietitian	Fat	2155	-0.231	[-0.429, -0.034]	0.216	0.002	0.178	no
Neutral dietitian	Carb	2155	-0.773	[-1.1, -0.4]	4.1e-04	2.5e-07	0.136	no
Neutral dietitian	Salt	2155	-0.024	[-0.041, -0.007]	0.068	2.3e-04	0.422	no
US dietitian	Energy	2156	-3.6	[-6.8, -0.4]	0.216	0.001	0.032	no
US dietitian	Protein	2156	+0.172	[-0.005, +0.349]	0.293	0.227	0.118	no
US dietitian	Fat	2156	-0.239	[-0.451, -0.027]	0.216	6.9e-04	0.125	no
US dietitian	Carb	2156	-0.422	[-0.842, -0.002]	0.293	0.002	0.007	no
US dietitian	Salt	2156	-0.061	[-0.080, -	3.5e-08	1.3e-13	0.995	yes

Condition	Nutrient	n	Mean shift	95% CI	p (Holm)	p (Wilcoxon)	Share significant (1,000 re-assignments)	Robust
				0.041]				
US homemaker	Energy	2155	-1.3	[-4.3, +1.8]	1.000	0.185	0.009	no
US homemaker	Protein	2155	-0.006	[-0.183, +0.172]	1.000	0.077	0.001	no
US homemaker	Fat	2155	+0.008	[-0.209, +0.226]	1.000	0.412	0.002	no
US homemaker	Carb	2155	-0.435	[-0.807, -0.062]	0.216	0.013	0.043	no
US homemaker	Salt	2155	-0.052	[-0.070, -0.033]	6.4e-07	8.9e-14	0.992	yes
smaller	Energy	2156	-51.7	[-55.9, -47.6]	4.2e-115	2.0e-137	1.000	yes
smaller	Protein	2156	-2.2	[-2.4, -1.9]	5.8e-76	3.4e-102	1.000	yes
smaller	Fat	2156	-2.6	[-2.9, -2.4]	1.4e-81	8.1e-103	1.000	yes
smaller	Carb	2156	-5.4	[-5.9, -4.9]	6.3e-92	2.7e-123	1.000	yes
smaller	Salt	2156	-0.201	[-0.222, -0.181]	6.3e-75	2.0e-92	1.000	yes
larger	Energy	2155	+108.6	[+102.0, +115.2]	2.2e-185	1.4e-209	1.000	yes
larger	Protein	2155	+4.8	[+4.4, +5.1]	3.7e-127	1.5e-166	1.000	yes
larger	Fat	2155	+5.6	[+5.2, +6.0]	7.3e-126	7.5e-165	1.000	yes

Condition	Nutrient	n	Mean shift	95% CI	p (Holm)	p (Wilcoxon)	Share significant (1,000 re-assignments)	Robust
larger	Carb	2155	+9.9	[+9.1, +10.6]	2.6e-139	7.8e-161	1.000	yes
larger	Salt	2155	+0.315	[+0.285, +0.345]	1.6e-83	3.6e-98	1.000	yes

Table S6. Pearson correlations with ground truth under the randomized model-assignment design

r (two-sided p, n) per condition × nutrient on the fixed random assignment, as annotated in Figure 7 of the main text. At n ≈ 2,157 every correlation is significant (all $p < 10^{-93}$; weakest cell Salt × larger, 8×10^{-94}); values are reported descriptively, and inferential claims rest on the Holm-corrected paired effect sizes (Figure 6, Table S5).

Condition	Energy	Protein	Fat	Carb	Salt
Baseline (none)	0.660 (3.1e-270, 2156)	0.688 (3.9e-302, 2156)	0.582 (1.6e-195, 2156)	0.629 (5.1e-238, 2156)	0.477 (4.5e-123, 2155)
JP dietitian	0.686 (4.8e-300, 2158)	0.695 (1.2e-311, 2158)	0.609 (5.3e-219, 2158)	0.657 (1.8e-266, 2158)	0.495 (7.3e-134, 2157)
JP homemaker	0.675 (1.5e-287, 2158)	0.693 (1.3e-308, 2158)	0.587 (2.5e-200, 2158)	0.646 (8.3e-255, 2158)	0.479 (2.3e-124, 2157)
Neutral dietitian	0.663 (7.8e-274, 2157)	0.689 (2.6e-303, 2157)	0.576 (2.7e-191, 2157)	0.628 (5.0e-237, 2157)	0.477 (7.5e-123, 2156)
smaller	0.652 (8.2e-262, 2159)	0.668 (1.3e-279, 2159)	0.578 (2.0e-192, 2159)	0.622 (1.3e-231, 2159)	0.483 (8.2e-127, 2158)
larger	0.575 (6.4e-190, 2158)	0.618 (1.0e-227, 2158)	0.499 (2.0e-136, 2158)	0.587 (1.2e-199, 2158)	0.422 (8.3e-94, 2157)
US dietitian	0.657 (4.8e-267, 2158)	0.676 (2.1e-288, 2158)	0.565 (2.7e-182, 2158)	0.621 (2.6e-230, 2158)	0.485 (1.1e-127, 2157)
US homemaker	0.646 (8.8e-255, 2157)	0.671 (2.9e-282, 2157)	0.555 (1.2e-174, 2157)	0.616 (3.5e-225, 2157)	0.485 (1.6e-127, 2156)

Table S7. Ground-truth distributions and composition profiles of the two datasets (complements main-text Tables 1 and 2)

Upper block: released ground-truth sets (SNAPMe n = 1,478 meals; NutriImage n = 892 dishes), including total portion weight (g). Lower block: evaluation sets (1,272 / 887), with the baseline bias (none condition, L0; mean of predicted − ground truth pooled over the six models).

Released sets (from the ground-truth files):

dataset	variable	n	median	q1	q3	mean	min	max
snapme	weight_g	1478	240.0	122.8	372.0	272.2	2.0	1570.5
snapme	energy_kcal	1478	243.4	94.6	437.8	301.0	0.0	1716.7
snapme	protein_g	1478	8.1	1.5	20.7	13.9	0.0	113.7
snapme	fat_g	1478	8.9	1.6	20.1	13.2	0.0	111.0
snapme	carb_g	1478	27.0	11.5	46.7	32.4	0.0	204.4
snapme	salt_g	1478	0.8	0.1	2.3	1.4	0.0	10.7
nutriimage	weight_g	892	130.0	81.0	213.0	151.7	10.0	499.8
nutriimage	energy_kcal	892	168.2	85.8	318.7	224.8	0.0	1073.0
nutriimage	protein_g	892	7.3	3.1	15.5	9.8	0.0	39.3
nutriimage	fat_g	892	6.6	2.1	15.4	9.7	0.0	72.8
nutriimage	carb_g	892	13.6	6.1	28.0	23.3	0.0	138.9
nutriimage	salt_g	891	1.2	0.5	1.9	1.5	0.0	47.4

Evaluation sets (with baseline bias):

dataset	nutrient	n	median	q1	q3	baseline bias (mean)
NI	energy_kcal	887	168.12	86.14	316.99	35.12
NI	protein_g	887	7.29	3.07	15.4	3.29
NI	fat_g	887	6.61	2.08	15.19	2.19
NI	carb_g	887	13.61	6.1	28.0	2.39
NI	salt_g	886	1.16	0.54	1.85	-0.26
SN	energy_kcal	1272	272.95	105.02	452.59	-12.82
SN	protein_g	1272	9.03	1.93	22.0	-1.61
SN	fat_g	1272	9.99	2.1	21.0	-1.1
SN	carb_g	1272	28.56	13.47	49.27	0.89
SN	salt_g	1272	0.92	0.13	2.42	-0.66

Per-category ground-truth nutrient profiles, NutriImage (detailed 12-category heuristic classification from dish names; per-dish mapping released with the data; mean ± SD):

Category	n	Energy (kcal)	Protein (g)	Fat (g)	Carb (g)	Salt (g)
Fish dishes	148	239 ± 151	14.5 ± 7.6	12.9 ± 11.2	14.4 ± 14.9	1.5 ± 1.4
Meat dishes	136	293 ± 152	14.0 ± 8.0	17.1 ± 10.1	19.3 ± 14.5	1.9 ± 4.1
Egg / tofu dishes	59	195 ± 128	10.5 ± 5.5	10.2 ± 6.8	14.4 ± 19.8	1.7 ± 1.4
Other protein	32	206 ± 145	8.9 ± 7.1	10.5 ± 8.6	18.1 ± 17.3	1.2 ± 1.2
Vegetable side dish	220	128 ± 135	5.3 ± 5.9	5.9 ± 8.3	13.3 ± 14.2	1.2 ± 1.9
Rice dishes	60	479 ± 241	13.2 ± 8.0	10.7 ± 10.4	78.7 ± 35.6	1.7 ± 1.5
Noodle dishes	47	459 ± 201	16.3 ± 8.7	10.6 ± 9.1	70.9 ± 26.8	3.5 ± 1.8
Bread / other staples	21	274 ± 131	9.1 ± 4.8	8.6 ± 5.2	39.4 ± 24.5	1.6 ± 1.4
Salad / pickle	58	105 ± 75	4.6 ± 3.6	5.6 ± 5.9	9.3 ± 5.8	1.0 ± 0.6
Soup / hot pot	31	143 ± 106	8.2 ± 7.1	5.9 ± 5.2	13.6 ± 11.4	2.0 ± 1.5
Dessert / fruit / snack	45	141 ± 89	3.1 ± 4.7	3.4 ± 5.8	25.0 ± 16.0	0.2 ± 0.4
Beverage	30	145 ± 101	4.1 ± 4.5	4.8 ± 6.3	18.6 ± 13.3	0.7 ± 1.0

Per-occasion ground-truth nutrient profiles, SNAPMe (eating occasion from the benchmark metadata; 1,271 of 1,272 meals matched; mean ± SD):

Occasion	n	Energy (kcal)	Protein (g)	Fat (g)	Carb (g)	Salt (g)
Breakfast	417	278 ± 222	11.2 ± 11.4	12.3 ± 13.8	32.1 ± 26.1	1.1 ± 1.4
Lunch	275	397 ± 281	20.4 ± 18.4	18.4 ± 17.7	39.1 ± 27.8	2.2 ± 1.8
Dinner	324	436 ± 256	23.6 ± 19.6	19.4 ± 15.2	41.6 ± 28.1	2.5 ± 1.8
Snack	206	171 ± 144	3.9 ± 5.3	6.6 ± 7.8	25.5 ± 22.2	0.4 ± 0.7
Drink only	49	64 ± 83	1.0 ± 1.9	1.3 ± 3.3	8.4 ± 11.7	0.1 ± 0.1

Food-group composition of the SNAPMe meals (ASA24 food groups from the benchmark metadata [2]): grains present in 55% of meals, protein foods 53%, vegetables 44%, dairy 43%, fruits 27%; median 2 food items per meal (IQR 1–4). The most frequent individual foods are everyday items (brewed coffee, raw tomatoes, bananas, olive oil, white rice, eggs,

sandwich breads, salad greens), indicating ordinary home-prepared or ready-made meals rather than restaurant-professional dishes.

Table S8. Equivalence (TOST) of pooled MedAPE under each condition (randomized-assignment sample)

Change in pooled MedAPE relative to baseline (percentage points) per condition × nutrient on the randomized-assignment sample (fixed assignment; bootstrap 90% CI over 2,000 image resamples; equivalence margin ±5 points; non-inferiority bound +5 points). Point estimates were confirmed stable across 300 independent re-assignments. Summary: non-inferiority in 25/25 persona-nutrient cells; two-sided equivalence in 22/25 (exceptions JP dietitian × energy/protein/carbohydrate, improvement side only); "smaller" 5/5 equivalent; "larger" fails non-inferiority in all four caloric nutrients.

condition	nutrient	n	dMedAPE	ci90_lo	ci90_hi	equivalent
jp	energy_kcal	2151	-3.73	-5.57	-1.94	False
jp	protein_g	2131	-3.95	-5.72	-2.36	False
jp	fat_g	2106	-2.56	-4.79	-0.79	True
jp	carb_g	2122	-4.15	-6.24	-2.04	False
jp	salt_g	2089	-1.38	-2.94	0.28	True
jp_home	energy_kcal	2151	-2.9	-4.69	-1.16	True
jp_home	protein_g	2131	-1.72	-3.49	-0.31	True
jp_home	fat_g	2106	-1.99	-4.24	-0.07	True
jp_home	carb_g	2122	-1.57	-3.72	0.24	True
jp_home	salt_g	2089	-1.1	-2.49	0.43	True
neutral_dietitian	energy_kcal	2151	-1.96	-3.45	-0.34	True
neutral_dietitian	protein_g	2131	0.03	-1.4	1.34	True
neutral_dietitian	fat_g	2106	-1.58	-3.86	-0.09	True
neutral_dietitian	carb_g	2122	-0.37	-2.15	1.4	True
neutral_dietitian	salt_g	2089	-0.74	-2.21	0.69	True
us	energy_kcal	2152	-1.75	-3.12	-0.14	True
us	protein_g	2132	-1.39	-3.14	0.2	True
us	fat_g	2107	-0.36	-2.4	1.62	True
us	carb_g	2123	-1.38	-2.81	1.14	True
us	salt_g	2090	-0.24	-1.76	1.2	True
us_home	energy_kcal	2151	-1.6	-2.92	0.32	True
us_home	protein_g	2131	0.86	-0.67	2.24	True
us_home	fat_g	2106	1.23	-0.73	3.31	True

condition	nutrient	n	dMedAPE	ci90_lo	ci90_hi	equivalent
us_home	carb_g	2122	-1.0	-2.63	0.9	True
us_home	salt_g	2089	-0.6	-1.9	0.74	True
smaller	energy_kcal	2152	-1.26	-3.0	0.96	True
smaller	protein_g	2132	-2.69	-4.49	-0.9	True
smaller	fat_g	2107	-1.69	-4.02	0.22	True
smaller	carb_g	2123	-1.42	-3.85	0.64	True
smaller	salt_g	2090	2.38	0.92	3.98	True
larger	energy_kcal	2151	19.23	16.52	21.87	False
larger	protein_g	2131	19.22	15.94	21.99	False
larger	fat_g	2106	20.61	16.59	23.99	False
larger	carb_g	2122	18.72	15.2	21.51	False
larger	salt_g	2089	2.25	0.2	3.9	True

Pooled energy residual summary per condition (fixed assignment; mean, SD, MAE in kcal):

Condition	Residual mean	Residual SD	MAE	n
none (baseline)	+10.3	181.4	122.4	2156
JP dietitian	−15.1	174.0	113.1	2155
JP homemaker	−9.3	177.4	114.1	2155
Neutral dietitian	+4.2	180.2	119.6	2155
US dietitian	+6.7	183.5	122.6	2156
US homemaker	+9.0	185.7	122.7	2155
smaller	−41.4	177.9	119.2	2156
larger	+118.9	247.3	190.0	2155

Figure S1. Sample-size flow

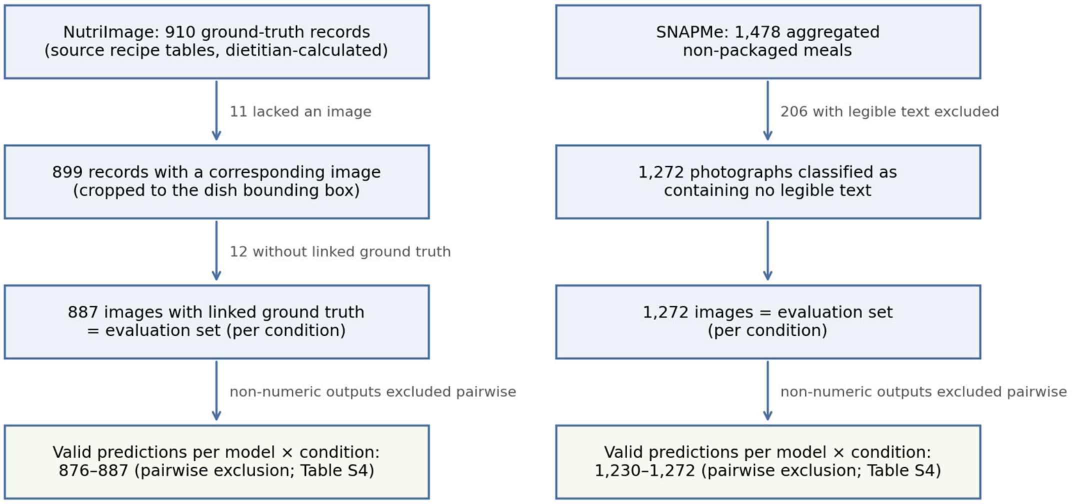

Figure S1. Sample-size flow for the two datasets, from source records to the evaluation sets and per-condition valid predictions (Table S4).

Figure S2. Per-model median shift curves

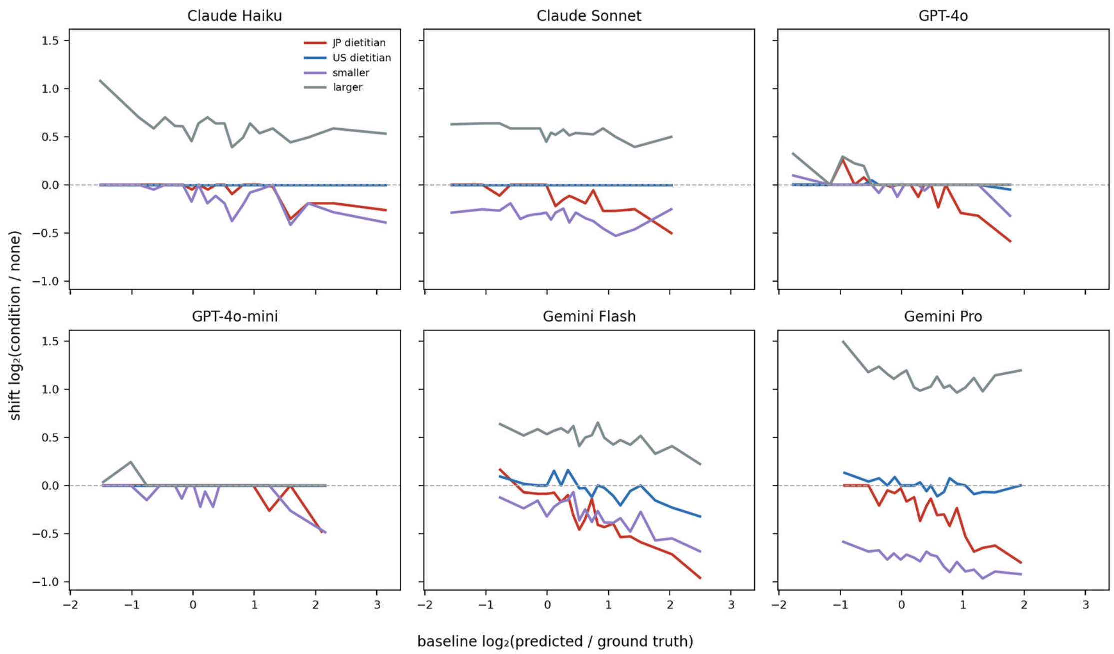

Figure S2. Per-model running-median shift curves (NutriImage, L0, energy): per-image shift $\log_2$(condition/none) versus per-image baseline $\log_2$(predicted/ground truth), running medians over 20 quantile bins, for the JP-dietitian, US-dietitian, smaller, and larger

conditions. The conditional character of the JP shift (near zero for under-estimated images, deepening with overestimation) reproduces in every model, while the explicit instructions produce approximately constant offsets.

Figure S3. Per-model, per-nutrient predicted versus ground truth (NutriImage)

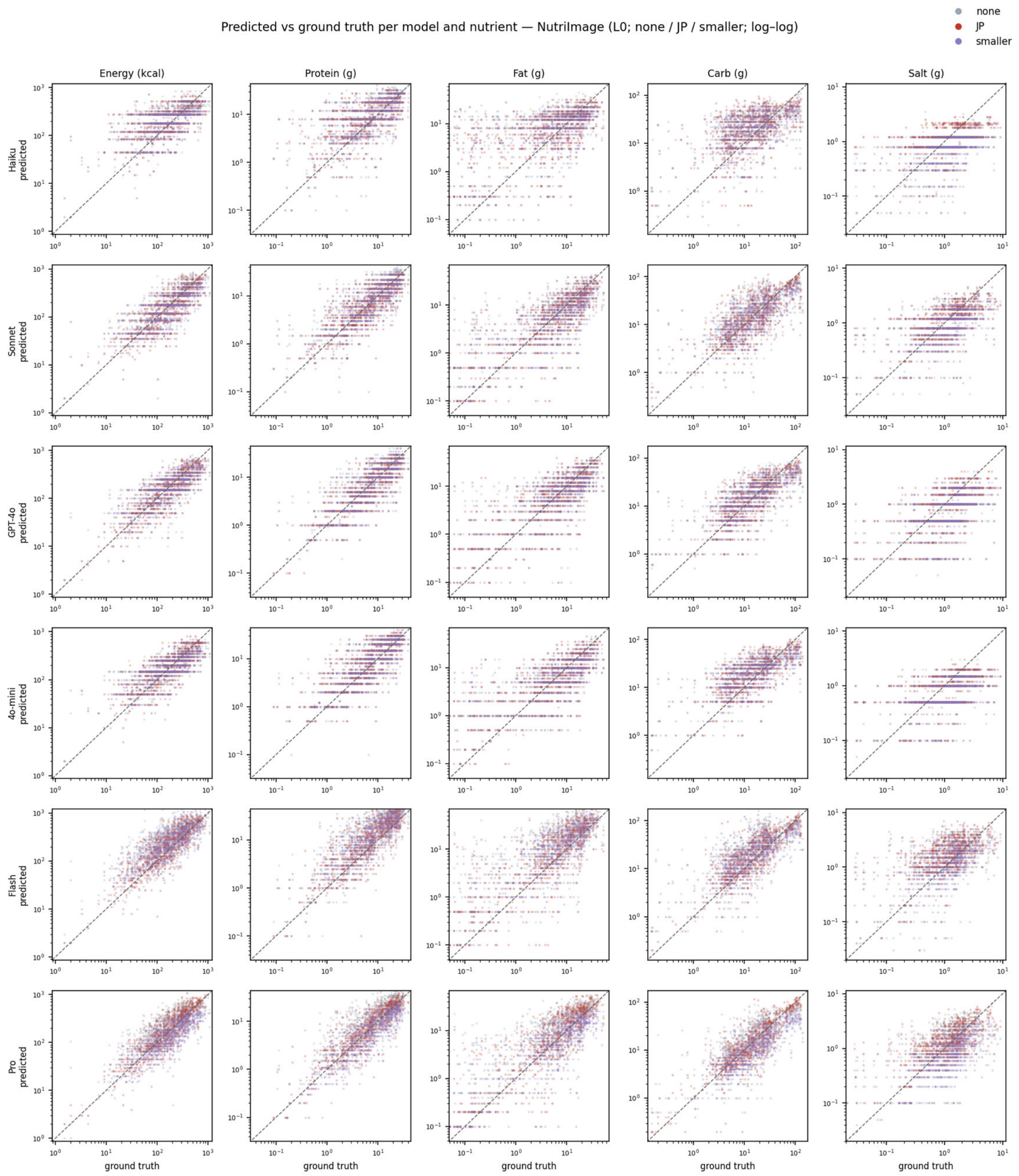

Figure S3. Predicted versus ground-truth values (log–log) per model × nutrient on NutriImage (L0), overlaying the none, JP-dietitian, and smaller conditions. Dashed: identity.

Figure S4. Per-model, per-nutrient predicted versus ground truth (SNAPMe)

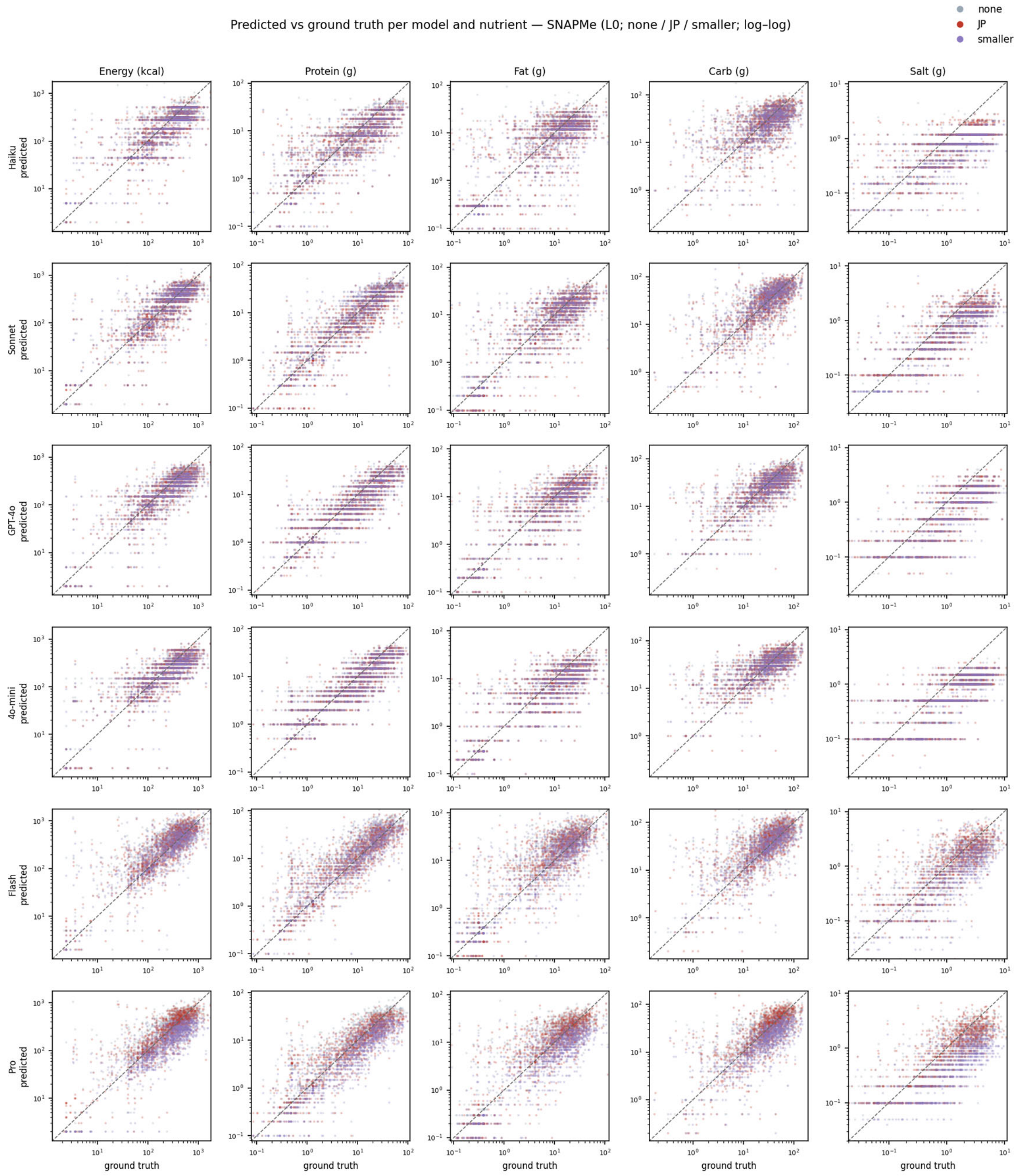

Figure S4. As Figure S3, for SNAPMe.
